# High throughput resistance profiling of *Plasmodium falciparum* infections based on custom dual indexing and Illumina next generation sequencing-technology

**DOI:** 10.1038/s41598-017-02724-x

**Published:** 2017-05-25

**Authors:** Sidsel Nag, Marlene D. Dalgaard, Poul-Erik Kofoed, Johan Ursing, Marina Crespo, Lee O’Brien Andersen, Frank Møller Aarestrup, Ole Lund, Michael Alifrangis

**Affiliations:** 1 Centre for Medical Parasitology, Department of International Health, Immunology and Microbiology, University of Copenhagen, 1356 Copenhagen K, Denmark; 2 Department of Infectious Diseases, Copenhagen University Hospital, 2200 Copenhagen N, Denmark; 3 Department of Systems Biology, Technical University of Denmark, Kemitorvet Building 208, 2800 Kgs. Lyngby, Denmark; 4 Department of Paediatrics, Kolding Hospital, University of Southern Denmark, 6000 Kolding, Denmark; 5 Bandim Health Project, Bissau, Guinea-Bissau; 6 Department of Microbiology, Tumor and Cell Biology, Karolinska Institutet, Stockholm, Sweden; 7 Department of Microbiology and Infection Control, Statens Serum Institut, 2300 Copenhagen S, Denmark; 8 National Food Institute, Technical University of Denmark, 2800 Kgs. Lyngby, Denmark

## Abstract

Genetic polymorphisms in *P. falciparum* can be used to indicate the parasite’s susceptibility to antimalarial drugs as well as its geographical origin. Both of these factors are key to monitoring development and spread of antimalarial drug resistance. In this study, we combine multiplex PCR, custom designed dual indexing and Miseq sequencing for high throughput SNP-profiling of 457 malaria infections from Guinea-Bissau, at the cost of 10 USD per sample. By amplifying and sequencing 15 genetic fragments, we cover 20 resistance-conferring SNPs occurring in *pfcrt*, *pfmdr1*, *pfdhfr*, *pfdhps*, as well as the entire length of *pfK13*, and the mitochondrial barcode for parasite origin. SNPs of interest were sequenced with an average depth of 2,043 reads, and bases were called for the various SNP-positions with a p-value below 0.05, for 89.8–100% of samples. The SNP data indicates that artemisinin resistance-conferring SNPs in *pfK13* are absent from the studied area of Guinea-Bissau, while the *pfmdr1* 86 N allele is found at a high prevalence. The mitochondrial barcodes are unanimous and accommodate a West African origin of the parasites. With this method, very reliable high throughput surveillance of antimalarial drug resistance becomes more affordable than ever before.

## Introduction

During the past fifteen years the global malaria case numbers are estimated to have decreased by 18% and overall death rates by 48%^1^. According to the WHO, fast-acting and efficacious antimalarial treatment is a key contributor to this development^1^. A major challenge in the further battle against malaria is therefore the persistent development and geographical spread of antimalarial drug resistance^2^. Unfortunately, tolerance and/or resistance has emerged towards all of the existing antimalarial treatments, and some areas struggle with highly multi-resistant parasites that represent a major threat to the rest of the malaria-endemic world^3^.

The current first-line treatments for *P. falciparum* malaria are the artemisinin-based combination therapies (ACTs). These are based on artemisinin derivatives^4^ combined with a partner drug: either amodiaquine (AQ), mefloquine (MQ), lumefantrine (LMF), piperaquine (PPQ) or sulphadoxine-pyrimethamine (SP)^1^. Due to the previous use of all of the partner drugs, except for LMF, as monotherapies in treating malaria, the parasites may already have developed tolerance/resistance towards these drugs. Furthermore, despite committed implementation of ACTs in all sub-Saharan African (SSA) countries, household surveys have estimated that quinine (QN) as well as abandoned drugs for treatment of malaria, namely chloroquine (CQ) and SP still account for approximately half of the antimalarial treatments given to children in SSA in 2014^1^. Lastly, two strategies of SP-based preventive treatment; intermittent preventive treatment of pregnant women (IPTp) and seasonal malaria chemoprevention (SMC, combining SP with AQ) are implemented in a number of countries^1^. *P. falciparum* parasites are therefore still under selective pressure from a wide range of antimalarial drugs.

Among the *P. falciparum* genes hitherto implicated in conferring antimalarial resistance, the literature has focused on single nucleotide polymorphisms (SNPs) occurring in mainly five *P. falciparum* genes; *P. falciparum chloroquine resistance transporter* (*pfcrt)*, *P. falciparum multidrug resistance gene 1 (pfmdr1), P. falciparum dihydrofolate reductase (pfdhfr)*, *P. falciparum dihydropteroate synthase* (*pfdhps)*, and most recently *P. falciparum putative kelch protein* (*pfK13)* ^5–20^. While specific and highly conservative SNPs have been identified globally in *pfcrt*, *pfmdr1*, *pfdhfr* and *pfdhps*, studies indicate that tolerance/resistance towards artemisinins may rather be conferred by a variety of mutations in the propeller region of *pfK13* ^15, 21^. So far 13 singly occurring mutations in the propeller region of *pfK13* have been identified in SE Asia and confirmed to mediate slow clearance time of the parasites. However, numerous low frequency *pfK13* polymorphisms, especially synonymous ones, have been identified in Africa, and are hitherto not linked to artemisinin resistance. Temporal surveillance of the prevalence of these SNPs provides crucial knowledge on development and geographic spread of antimalarial drug resistance in *P. falciparum* populations, which are important for antimalarial drug policy decisions for instance as shown regarding IPTp^22^.

The geographic origin of the parasites is of major importance to a surveillance scheme aiming to monitor global emergence and spread of resistance, and perhaps thereby attempt to hinder or delay the spread of artemisinin resistance from South East Asia to SSA^23–25^. According to a recently published study, recent geographic origin of the parasites can be estimated with a genetic barcode consisting of well-defined SNPs located in the mitochondrial and apicomplexan genomes^26^. A surveillance scheme aiming to analyse all SNPs in question is therefore a long process and requires sequencing.

The knowledge thus exists for temporal and geographical surveillance of antimalarial drug resistance including geographic flow of parasites between continents. For such a surveillance scheme to be operational, it requires high throughput SNP-profiling through sequencing, while allowing for identification and grouping of the data pertaining to an individual infection. A number of methods have been developed for SNP-detection, including PCR-RFLP, PCR-SSOP-ELISA, real-time PCR, LAMP and custom DNA micro-arrays^27–39^, however, only few of them are suitable for large-scale screening. Methods that allow for actual fragment sequencing are limited to Sanger sequencing and next-generation sequencing (NGS), where NGS in particular is highly suited for high throughput methodologies. So far, two NGS-based methods of relevance for surveillance of antimalarial drug resistance have been published^40, 41^, whereof only one allows for barcoding and thereby track-keeping of sequences pertaining to a specific infection^41^.

In this study, we present proof-of-concept of using custom dual indexing on the Illumina NGS (Miseq) platform, for targeted high throughput sequencing of genetic loci related to antimalarial drug resistance and mitochondrial barcoding of the infecting *P. falciparum* parasites. Application of an NGS platform vastly increases the amount of raw data acquirable as compared to previously applied methods, and thereby provides the opportunity for implementation of a high throughput methodology. The Illumina platform described in the present study is designed for dual indexing of samples, which consists of a 3′ and 5′ individual barcode, which allow the user to connect every sequence generated to a sample (infection) of origin. In our method, we have applied custom dual indexing based on primers that were custom-designed, which allows for a substantial increase in barcode combinations and therefore in the number of samples that can be run simultaneously.

## Results

**Figure 1:**
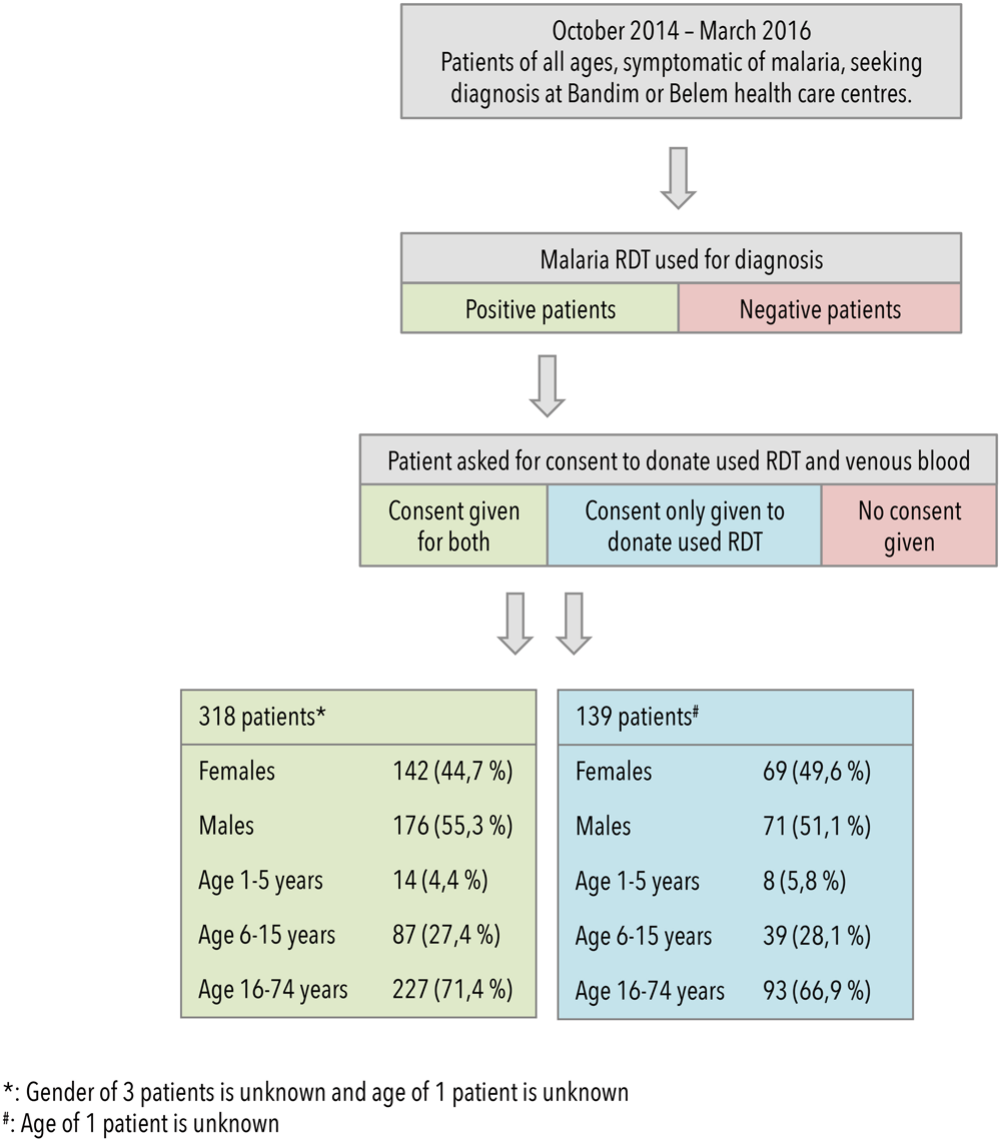
Patient recruitment and demography. Patients of all ages attending the Bandim or Belem health care centres, with a fever or a history of fever within the past 24 hours, were tested for malaria positivity with an RDT. If the patient tested positive, the patient was asked for informed consent in donating the RDT used to test them for malaria infection. The malaria-positive patients were furthermore asked for informed consent in donating venous blood. If consent was given for this procedure, the blood was drawn and the patient was included in the study. In total, 457 malaria-positive patients gave consent to donate their RDTs and 318 of these patients gave consent to donate venous blood. If venous blood was donated, the RDT was not used for the analysis.

A total of 457 patients of all ages attending the Bandim and Belem Health Care Centres in Guinea-Bissau, were diagnosed with malaria with an RDT, and donated their used RDTs to the current study. Additionally, out of these patients, 318 patients gave consent to donate venous blood, which was processed and dried on filter paper (see methods). See Fig. 1 for sampling overview and demographic details. The 318 patients who donated filter paper samples were also diagnosed by microscopy. Parasite densities ranged from 15 parasites/200 leucocytes to 500 parasites/13 leucocytes. Filter paper samples were used for full resistance profiling and mitochondrial barcoding while RDTs were only used for mitochondrial barcoding.

### Sample calling and sensitivity

**Figure 2:**
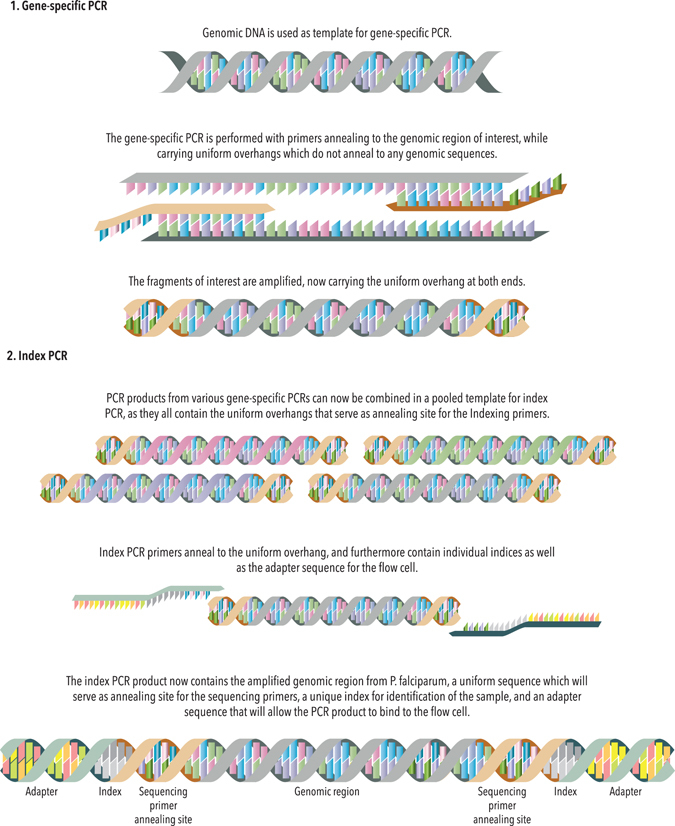
Primer and fragment design for library preparation. The *P. falciparum* gene fragments of interest are amplified with gene-specific primers, in a “gene-specific PCR” (1). The primers anneal to loci in the *P. falciparum* genome, while encoding non-annealing overhangs, which are then incorporated into the PCR products during amplification. The non-annealing overhangs are identical on all gene-specific primers, and serve as annealing site for the index primers (2), which can in turn anneal to all amplified gene fragments. The index primers also encode individual 8-base indices as well as an adaptor sequence. The index and adapter sequence are incorporated into the PCR product (denoted amplicon from here on), which can then bind to the Miseq flow cell through the adaptor sequence, and be identified through sequencing of it’s unique index combination.

Several steps in the protocol of the platform presented in this study have been optimised for analysis of large numbers of samples. Multiplexing of the gene-specific PCRs followed by further multiplexing of the index PCRs and finally pooling of samples prior to sequencing (see Figs 2 and S1 for details), are all elements that meet the necessity of efficiency in high throughput surveillance, but which reduce the possibility of keeping track of all the individual PCR fragments and samples in the analysis. It is therefore crucial to investigate to what extent individual amplicons and samples were lost during the process. All of the 463 custom dual index combinations (457 samples and 6 controls) were successfully called from the sequencing data with belonging amplicon sequences, and hence no samples had been completely lost, however some had too few reads for SNP-analysis. Out of the 318 samples that were resistance-profiled, 264 were successfully sequenced in all resistance-conferring positions. Of the 54 samples that were not completely profiled, 45 were lacking adequate sequencing of a single amplicon, 7 were lacking adequate sequencing of 2 amplicons, while 2 samples were lacking adequate sequencing of 3 amplicons. Mitochondrial barcodes were successfully generated for all 457 samples. There was no correlation between parasite density and successful resistance-profiling/amplicon coverage.

### Amplicon representation and coverage

**Figure 3:**
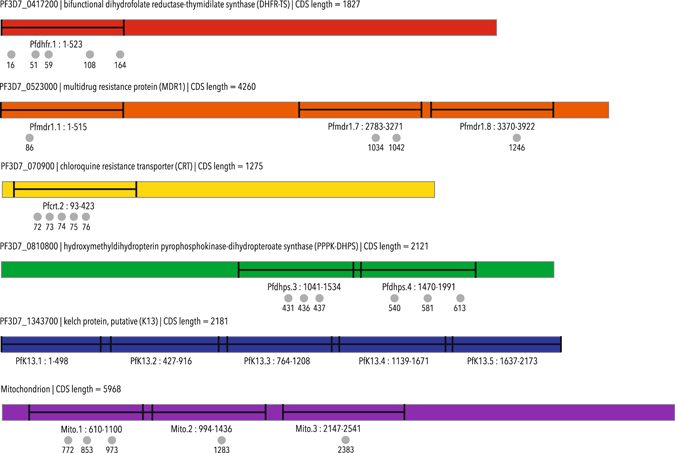
Amplicon design. In the current study, we have analysed one fragment including five SNPs in *pfdhfr*, three fragments including four SNPs in *pfmdr1*, one fragment including five SNPs in *pfcrt*, two fragments including six SNPs in *pfdhps*, all of *pfK13*, and three fragments including five SNPs in the mitochondrial genome of *P. falciparum*. The figure illustrates the length and placement of the fragments in the different genes, as well as the nucleotide/codon positions of the SNPs of interest.

The various amplicons sequenced in this study are depicted in Fig. 3. Amplicon representation was defined as the presence of sequences corresponding to a specific amplicon within the individual sample. Base calling was defined as the determination of a base at a specific position within the amplicon, and was also assessed for individual samples. Both amplicon representation and successful base calling for the individual samples was assessed after performing quality trimming of the raw sequences (see methods). The amplicon representation (listed in Table 1) was estimated as a presence/non-presence of sequences belonging to a specific amplicon in the individual samples (read count >0) and ranged from 98.1% to 100%. Successful base calling (determined as bases called with a minimum z-score of 1.96 and p-value ≤ 0.05) was estimated for all surveyed SNP-positions, as well as positions from each mitochondrial amplicon, and ranged from 89.8% to 100%. Average read count for the different resistance-conferring SNPs (per sample) varied from 250 reads to 4,734 reads (mean = 1,520 reads for individual bases, and 1,725 reads for entire amplicons, Table 1). Average read count over all amplicons was 2,043.

**Table 1:** Amplicon Amplicon representation, successful base calling and average read count.

PCR product	CODON	Amplicon representation		Successful base-calling		average read count
		count	percentage	count	percentage	
Pfcrt.2	72	324	100	320	98,8	1129
Pfcrt.2	73	324	100	320	98,8	1129
Pfcrt.2	74	324	100	320	98,8	1128
Pfcrt.2	75	324	100	320	98,8	1128
Pfcrt.2	76	322	99,4	320	98,8	1128
Pfdhfr.1	16	324	100	323	99,7	2463
Pfdhfr.1	51	324	100	323	99,7	2335
Pfdhfr.1	59	324	100	323	99,7	2324
Pfdhfr.1	108	324	100	322	99,4	2448
Pfdhfr.1	164	324	100	323	99,7	2385
Pfdhps.3	431	324	100	322	99,4	774
Pfdhps.3	436	324	100	322	99,4	767
Pfdhps.3	437	324	100	322	99,4	767
Pfdhps.4	540	313	96,6	291	89,8	250
Pfdhps.4	581	320	98,8	311	96,0	1575
Pfdhps.4	613	320	98,8	311	96,0	1601
Pfmdr1.1	86	324	100	324	100	4734
Pfmdr.7	1034	318	98,1	307	94,8	440
Pfmdr.7	1042	318	98,1	307	94,8	440
Pfmdr.8	1246	324	100	322	99,4	1467
PfK13.1	84	324	100	324	100	2008
PfK13.2	224	322	99,4	320	98,8	755
PfK13.3	329	324	100	324	100	1511
PfK13.4	469	323	99,7	323	99,7	5963
PfK13.5	635	324	100	324	100	5816
Mito.1	base 772	324	100	324	100	2496
Mito.2	base 1283	324	100	324	100	3224
Mito.3	base 2383	324	100	324	100	5015

The table shows how many of the samples were found with pertaining reads corresponding to the various amplicons, after quality trimming (as sample count and percentage), as well as for how many samples successful base-calling was performed (requires a minimum z-score of 1.96). For *pfK13* and for the mitochondrion, a position for each amplicon was analysed. Some of the SNP positions are located in the same amplicon. The table also lists the name of the individual amplicons, and Fig. 3 can be referred to for illustrations of the length and position of the amplicons within the various genes.

### SNP-data from *P. falciparum* positive subjects attending the Bandim and Belem Health Care Centres in Guinea-Bissau

**Figure 4:**
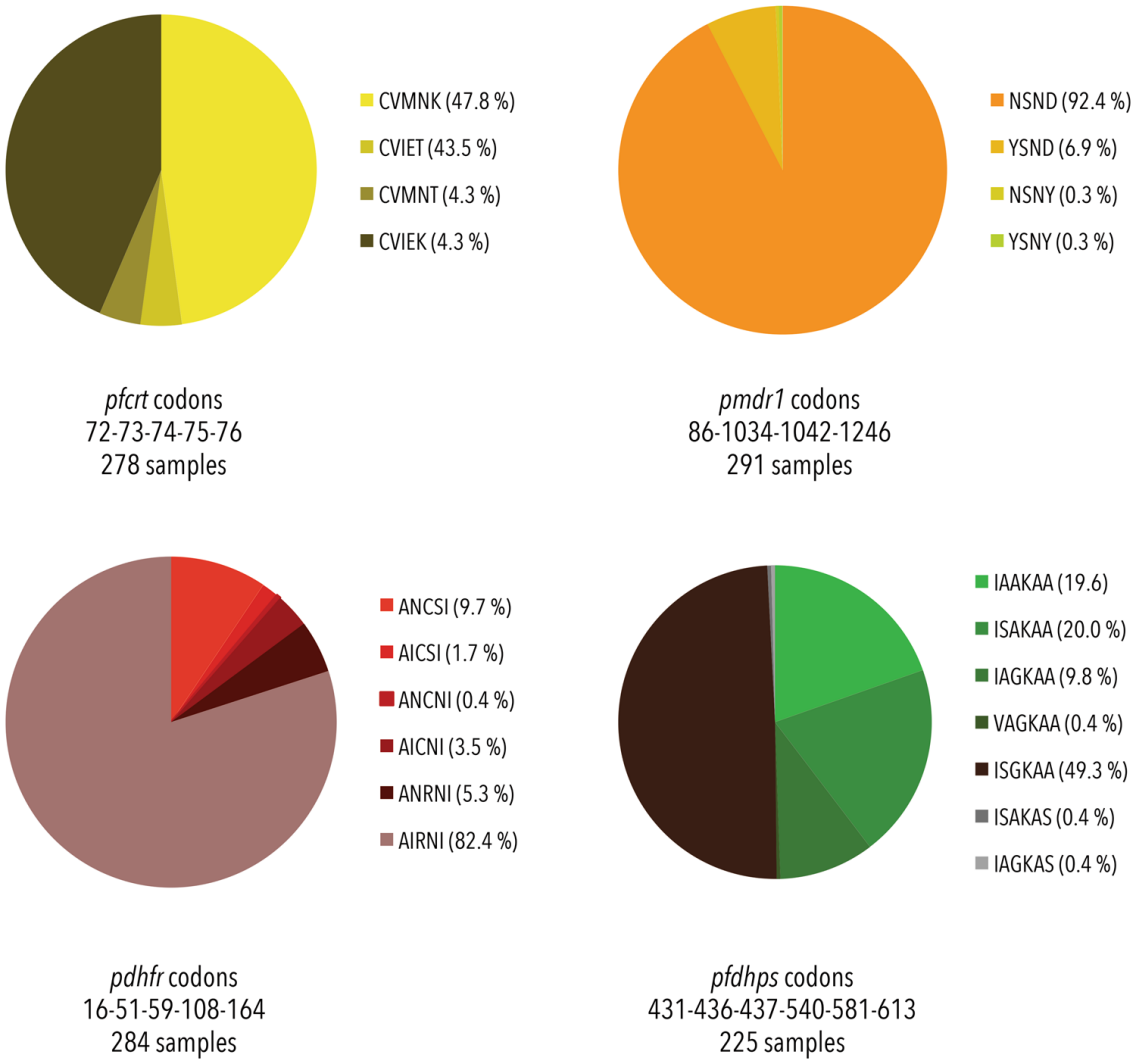
Haplotype distributions. Four genes were analysed for the distribution of different haplotypes, due to the relevance of certain haplotypes in conferring different levels of tolerance/resistance towards various antimalarial drugs. The figure illustrates the distribution of haplotypes (mixed infections omitted) found in *pfcrt*, *pfmdr1*, *pfdhfr* and *pfdhps*. Complete haplotypes could be assigned to 278 samples for *pfcrt*, 291 samples for *pfmdr1*, 284 samples for *pfdhfr* and 225 samples for *pfdhps*.

Table 2 lists the results of frequencies of known molecular markers in *pfcrt*, *pfmdr1*, *pfdhfr* and *pfdhps*. Samples with <75% unanimous reads were categorized as mixed, and therefore counted in both the reference and alternative group. Furthermore, Table 3 lists other polymorphisms found (only *pfcrt* and *pfmdr* were found to have additional polymorphisms) and Table 4 describes all polymorphisms found in *pfK13*. The data acquired for the six control strains is listed in Table 5. Finally, Fig. 4 illustrates the distribution of the various haplotypes of *pfcrt*, *pfmdr*, *pfdhfr* and *pfdhps* (excluding mixed infections).

**Table 2:** Prevalence of resistance-conferring mutations.

	Reference codon	Alternative codon	Reference freq.	Alternative freq.	Mixed freq.	Total count
pfcrt_72	C	S	100.0%	0.0%	0.0%	314
pfcrt_73	V	—	100.0%	0.0%	0.0%	314
pfcrt_74	M	I	57.0%	52.9%	9.9%	314
pfcrt_75	N	E	57.0%	52.9%	9.9%	314
pfcrt_76	K	T	56.5%	52.4%	9.8%	317
pfdhfr_16	A	V	100.0%	0.0%	0.0%	317
pfdhfr_51	N	I	18.6%	88%	6.6%	317
pfdhfr_59	C	R	18.9%	87.1%	6.3%	317
pfdhfr_108	S	N	12.6%	92.4%	4.7%	316
pfdhfr_164	I	L	100.0%	0.0%	0.0%	317
pfdhps_431	V	I	99.4%	0.6%	0.0%	316
pfdhps_436	A	S	74.3%	35.7%	10.1%	316
pfdhps_437	A	G	50.3%	66.8%	17.1%	316
pfdhps_540	K	E	100.0%	0.3%	0.3%	286
pfdhps_581	A	G	100.0%	0.0%	0.0%	306
pfdhps_613	A	S/T	99.3%	0.7%	0.0%	306
pfmdr1_86	N	Y	92.8%	9.7%	2.5%	318
pfmdr1_1034	S	C	100.0%	0.0%	0.0%	301
pfmdr1_1042	N	D	99.7%	0.3%	0.0%	301
pfmdr1_1246	D	Y	99.0%	1.0%	0.0%	313

The table describes what frequencies of the samples that were found to harbor the reference SNPs versus the alternative SNPs (translated into codons) and how many of the samples were categorized as mixed infections. Note that mixed infections (noted as Mixed freq.) were counted in both the reference and alternative frequencies. The analysis was done for every SNP-position of interest in *pfcrt*, *pfmdr1*, *pfdhfr* and *pfdhps*.

**Table 3:** Other polymorphisms in *pfcrt* and *pfmdr1*.

	Base position	Codon position	Reference base	Alternative base	Reference codon	Alternative codon	Reference freq.	Alternative freq.	Mixed freq.
**Non-synonymous polymorphisms**									
*pfcrt*	382	128	T	A	L	I	99.4%	1.3%	0.6%
*pfmdr1*	35	12	A	C	N	T	99.4%	0.6%	0.0%
	2903	968	G	T	G	V	99.7%	0.3%	0.0%
	3597	1199	T	G	N	K	95.0%	7.8%	2.8%
**Synonymous polymorphisms**									
*pfmdr1*	306	102	T	C	G	G	96.3%	5.6%	1.9%
	447	149	T	G	S	S	97.8%	2.2%	0.0%
	3186	1062	C	T	D	D	99.7%	0.3%	0.0%
	3207	1069	T	G	T	T	94.8%	7.2%	2.0%
	3411	1137	A	C	Y	Y	98.8%	1.6%	0.3%
	3417	1139	A	G	P	P	99.7%	0.6%	0.3%

The table describes polymorphisms identified amongst the 318 resistance-profiled samples in *pfcrt* and *pfmdr1* other than the positions of interest indicated on Fig. 3 and Table 2. The frequencies of both the reference and alternative are listed, as well as the frequency of mixed infections. Note that mixed infections were counted in both the reference and alternative frequencies.

**Table 4:** Polymorphisms in *pfK13*.

Base position	Codon position	Reference base	Alternative base	Reference codon	Alternative codon	Reference freq.	Alternative freq.	Mixed freq.
**Non-synonymous K13 polymorphisms**								
65	22	G	A	R	K	99.7%	0.3%	0.0%
252	84	A	G	L	K	99.7%	0.3%	
335	112	G	A	G	E	99.7%	0.9%	0.6%
340	114	A	C	L	Q	99.1%	1.3%	0.3%
445	149	A	T	T	S	99.4%	0.6%	
566	189	A	C	L	T	53.8%	57.9%	11.6%
567	189	A	T	L	N	98.1%	3.5%	1.6%
820	274	C	T	H	Y	99.7%	0.3%	
*1732*	578	G	T	A	S	99.1%	1.6%	0.6%
*2092*	698	T	A	F	I	99.7%	0.3%	
**Synonymous K13 polymorphisms**								
57	19	G	A	T	T	99.7%	0.3%	
321	107	T	C	S	S	99.7%	0.3%	
417	139	T	C	N	N	99.4%	0.9%	0.3%
*1407*	469	C	T	C	C	99.1%	1.9%	0.9%
*1434*	478	C	G	T	T	99.7%	0.3%	
*1881*	627	T	A	A	A	99.7%	0.3%	
*2070*	690	C	A	G	G	99.7%	0.3%	

The table describes the various polymorphisms found in *pfK13* for the 318 resistance-profiled samples, and the frequencies in which the reference and alternative base were found, as well as the frequencies of mixed infections. SNP positions written in italic indicate that they are located in the propeller region of *pfK13*. Note that mixed infections were counted in both the reference and alternative frequencies.

**Table 5:** Control data.

SNP	Control strains with listed codon/base (and read count)					
	3D7	7G8	DD2	K1	MRA_1238	MRA_1239
pfcrt_72	C (1238)	S (1752)	C (153)	C (2013)	C (1968)	C (1753)
pfcrt_73	V (1238)	V (1752)	V (153)	V (2013)	V (1968)	V (1753)
pfcrt_74	M (1238)	M (1752)	I (153)	I (2013)	I (1968)	I (1753)
pfcrt_75	N (1238)	N (1752)	E (153)	E (2013)	E (1968)	E (1753)
pfcrt_76	K (1238)	T (1752)	T (153)	T (2013)	T (1968)	T (1753)
pfdhfr_16	A (3744)	A (1954)	A (148)	A (1373)	A (2646)	A (1182)
pfdhfr_51	N (3561)	I (1837)	I (149)	N (1302)	I (2520)	I (1129)
pfdhfr_59	C (3532)	C (1823)	R (137)	R (1295)	R (2513)	R (1124)
pfdhfr_108	S (3721)	N (1935)	N (154)	N (1355)	N (2615)	N (1167)
pfdhfr_164	I (3607)	I (1901)	I (155)	I (1318)	I (2534)	L (1150)
pfdhps_431	I (568)	I (1126)	I (90)	I (5233)	I (1545)	I (7437)
pfdhps_436	S (565)	S (1119)	F (101)	S (5178)	A (1537)	S (7368)
pfdhps_437	G (565)	G (1120)	G (101)	G (5174)	G (1542)	G (7370)
pfdhps_540	K (75)	K (411)	K (88)	K (1196)	E (294)	Y (733)
pfdhps_581	A (403)	A (1045)	A (136)	G (3614)	A (828)	A (1878)
pfdhps_613	A (403)	A (1061)	S (135)	A (3730)	A (858)	A (1915)
pfmdr1_86	N (4543)	N (1350)	Y (540)	Y (4758)	N (6463)	N (6628)
pfmdr1_1034	S (555)	C (1417)	S (98)	S (1727)	S (988)	S (4078)
pfmdr1_1042	N (555)	D (1417)	N (98)	N (1727)	N (988)	N (4078)
pfmdr1_1246	D (3111)	Y (3189)	D (236)	D (1399)	D (3262)	D (6719)
pfK13_493	Y (3459)	Y (2389)	Y (172)	Y (1987)	H (3632)	Y (5430)
pfK13_543	I (4001)	I (3760)	I (267)	I (3801)	I (4099)	T (5698)
Mito_772	C (2977)	C (3666)	T (678)	T (452)	T (2496)	T (2590)

The table lists the acquired data for the control parasites. The data is listed as the acquired codon/base (only base for Mito_772, as the mitochondrial barcode is DNA-code based) followed by the amount of matching reads in parenthesis. None of the control samples were mixed genotypes.

#### Polymorphisms found in pfcrt

A fragment covering c42–131 of the *pfcrt* gene, was sequenced (Fig. 3). The c76 K/T alleles were present in almost equal amounts (including mixed K/T infections: 56.5% and 52.4%, respectively, Table 2), as was the case for the c72–76 haplotypes CVMNK/CVIET (47.8% and 43.5% respectively, mixed infections omitted, Fig. 4). Additionally, the CVIEK (n = 12) and the CVMNT (n = 12) were identified in few samples. Furthermore, a non-synonymous polymorphism at c128 (L to I) was found in four samples (1.3%), twice in mixed infections (Table 3).

#### Polymorphisms found in pfmdr1

In total, 1369 bases were sequenced in *pfmdr1*, covering c11–162, c938–1080, and c1134–1297 (Fig. 3). The *pfmdr1* c86N was found in 92.8% of the samples, while *pfmdr1* c86Y was found in only 9.7% of samples (Table 2). *pfmdr1* c184 was not analysed due to a design flaw. No samples were found to have mutations at *pfmdr1* c1034 and c1042, while only 2 samples had the *pfmdr1* c1246Y allele (Table 2). Additionally, non-synonymous mutations were found at c12 N→T (2 samples), c968 G→V (1 sample), and c1199 N→K (16 samples plus 9 mixed) (Table 3). Synonymous mutations were found at c102 (12 samples plus 6 mixed), c149 (7 samples), c1062 (1 sample), c1069 (16 samples plus 6 mixed), c1137 (4 samples plus 1 mixed) and c1139 (1 sample plus 1 mixed) (Table 3). A total of 262 samples were completely profiled in *pfcrt* and *pfmdr1*, and amongst these samples we found that 41.2% carried both the *pfcrt* c72–76 CVIET haplotype and the *pfmdr1* c86N allele.

#### Polymorphisms found in pfdhfr

A fragment of 461 bp was sequenced from *pfdhfr*, reaching from c11–164 (Fig. 3). The c16 A→V and the c164 I→L mutations were completely absent while the c51 N→I mutation was found in 88% of samples, the c59 C→R mutation was found in 87.1% of samples and the c108 S→N mutation was found in 92.4% of samples (Table 2). The prevalence of the *pfdhfr* triple mutant haplotype (51I, 59 R, 108 N) was 82.4% (mixed infections omitted, Fig. 4).

#### Polymorphisms found in pfdhps

Two fragments covering 887 bp reaching from c358–645 were sequenced for *pfdhps* (Fig. 3). The c431 V→I mutation was found in one sample, the c436 A→S polymorphism was found in 35.7% of samples, and the c437 A→G was found in 66.8% of samples. In contrary, the c540K→E mutation was only observed once in a mixed infection, the c581 A→G mutation was not observed at all and the c613 A→S was found once (Table 2). A total of 233 samples were completely profiled in *pfdhfr* and *pfdhps*, and amongst these samples we found that 52.8% carried the combined *pfdhfr/pfdhps* quadruple mutant (consisting of *pfdhfr* c51I, c59R, c108N + *pfdhps* c437G).

#### Polymorphisms found in pfK13

The entire length of *pfK13* (excluding forward primer site of amplicon K13.1 and reverse primer site of amplicon K13.5, see Fig. 3) was analysed for polymorphisms, and listed in Table 4. The c189T allele was found in 57.9% of the samples, while mixed c189L/T were found in 11.6% of samples. The c189N allele, was found in 3.5%, while both c189L/N were found in 1.6%. Other non-synonymous polymorphisms, of which 2/8 were located within the propeller region of *pfK13* (marked as blue in Table 3), were found at c22 R→K (1 sample), c84 L→K (1 sample), c112 G→E (1 sample plus 2 mixed), c114 L→Q (3 samples, 1 mixed), c149 T→S (2 samples), c274 H→Y (1 sample), c578 A→S (3 samples plus 2 mixed) and c698 F→I (1 sample). Also, a number of synonymous polymorphisms were found in *pfK13*, 4/7 of which were located in the propeller region: c19 (1 sample), c107 (1 sample), c139 (2 samples plus 1 mixed), c469 (3 samples plus 3 mixed), c478 (3 samples), c627 (1 sample) and c690 (1 sample).

#### Mitochondrial barcode

Mitochondrial barcodes were successfully provided for all 457 samples. The results revealed a unanimous pattern across all samples, namely the CTTAG barcode (corresponding to nucleotide positions 772-853-973-1283-2382), which corresponds to the barcodes published for West-Africa^26^.

## Discussion

Multi-drug resistant *P. falciparum* threatens the current trend of decreasing malaria burden observed in many malaria endemic countries. Molecular surveillance of antimalarial resistance is becoming an attractive operational tool to determine temporal and geographical emergence and spread of drug resistance. However, efficient molecular surveillance requires high throughput, sequence-based SNP analysis in order to assess all the SNPs and genes playing a role in antimalarial drug resistance. This study applied Illumina’s Miseq NGS technology of paired-end sequencing on samples collected from patients with *P. falciparum* infections to provide a high-throughput molecular surveillance method, which is cost-effective as compared to other published methodologies including classic Sanger sequencing.

The sequenced amplicons cover the most established molecular markers of drug resistance in *pfcrt, pfmdr1, pfdhfr* and *pfdhps*, as well as the entire *pfK13* gene. Additionally, the sequenced amplicons also covered the SNPs constituting the mitochondrial barcode linked to the geographical origin of *P. falciparum*. The method is PCR based and applies several PCR steps, ruling out the possibility of assessing gene copy numbers, as would be relevant for *pfmdr1* and the recently identified plasmepsin^42^. However, the number and identity of amplicons incorporated in the analysis is only dependent on primer design. The method is therefore highly adaptable, since polymorphisms for analysis can easily be incorporated, including for example the exonuclease E415G polymorphism, which has recently been identified as a marker for PPQ resistance^42^, *pfmdr1* c184, as well as SNPs occurring in the apicoplast, which can be used in further specifying the origin of the infecting parasite^42^. Importantly, the method is sequencing-based, which generates the possibility of monitoring entire genes, which is necessary in the case of polymorphisms in *pfK13* and their relation to artemisinin resistance. It also opens the possibility of identifying other polymorphisms that are not normally analysed with SNP-detection based methods, as is seen for *pfcrt* and *pfmdr1* in this study. The unique 5′- and 3′- indices enable identification of individual samples, and it is therefore feasible to analyse various genes belonging to the same infection, which again is necessary when monitoring parasite susceptibility to combination therapies, such as SP and ACTs.

The possibility of customising the library preparation is the reason the Illumina platform was chosen over other platforms that would provide longer sequences and hence require fewer amplicons for sequencing of entire genes. At 10 USD per sample, the current study is approximately 7x cheaper than an alternative protocol published for Ion Torrent^41^. We have created 50 unique indices (Table S2), which make it possible to create 2450 unique dual index combinations (50 × 50–50) and hence run as many samples on the Miseq simultaneously. It is thereby feasible to reduce sample costs to less than 4 USD per sample, approximating only 5 USD per sample already with only 800 samples. When running large numbers of samples simultaneously, the challenge consists of controlling the process of mixing all amplicons in equal amounts prior to sequencing (see Figure S1 for protocol overview and Figures S2 and S3 for example data). Targeting the desired depth of coverage of the various amplicons can be approximated through this process, also if certain amplicons are to be sequenced deeper than others. However, varying depth of coverage should be expected, especially if multiplex PCR is applied for gene amplification and subsequently during the library preparation, as is the case in our study. Sequencing bias may also occur when using the library design applied^43^, leading to vastly differentiated depth of coverage of the different amplicons. This is due to a potential mismatch between the Illumina Nextera sequencing primer and the designed amplicons, which can also occur when applying the Illumina Nextera Library Preparation Kit^43^. It is noteworthy, however, that despite such a bias, the final coverage can still be expected to be adequate, due to the mere sequencing capacity of the Illumina Miseq (amounting to 7.5 billion bp per run).

While gene haplotype analysis excludes analysis of mixed infections, frequencies of individual SNPs can be given for major and minor alleles as long as a minor allele is detectable. Since the aim of the current study was not only to provide frequency data, but also haplotype data, the cut off for mixed infections was set as <75% of the bases supporting a given base for a given position. This cut off, however, can be reduced and should be considered subjective to study purpose. The experimental limitations for the cut off are largely dependent on the achieved depth of coverage as well as the applied statistical probability applied for base calling.

The method applied in this study generated sequence data from *pfcrt, pfmdr1, pfdhfr, pfdhps* and *pfK13* from 318 *P. falciparum* samples. The polymorphisms discovered in *pfK13* in the current study indicate no *pfK13*-mediated artemisinin tolerance in Bandim, Guinea-Bissau. Accordingly, an efficacy study published in 2016 showed high efficacy of AL in the same area^3^. The majority of the non-synonymous polymorphisms found in *pfK13* were located in the N-terminal region (not the propeller), in particular the *pfK13* c189T was found at 57.9%. The high prevalence of this SNP is consistent with previous studies in Africa and it has not been associated with artemisinin resistance^15, 44, 45^. Only two non-synonymous polymorphisms were found in the propeller region of *pfK13*: c578S and c698I. The *pfK13* c578S has previously been identified at noteworthy frequencies in a number of SSA countries without clinical relevance^3, 15, 25, 46^. However, the data generated in this study cannot rule out any pfK13-independent mechanisms conferring resistance towards artemisinin, as well as the study cannot conclude on the treatment efficacy for the patients involved. Interestingly, a Kenyan study has recently published the presence of residual *P. falciparum* parasitemia at day 3 after treatment with either artemether-lumefantrine or dihydroartemisinin-piperaquine in 31.8% of the infected children^47^. A follow-up study furthermore rules out the responsibility of *PfK13-*encoded polymorphisms in in these day 3 positive cases^48^. Importantly, the mitochondrial barcodes indicated no recent import of *P. falciparum* parasites from South-East Asia, where artemisinin resistance is a reality. Mainly the first position in the barcode, base 772, would reveal recent South-East Asian origin, if it were a T instead of a C^26^. All four of the South-East Asian control parasites were found to have a T on position 772 (DD2, K1, MRA-1238, MRA-1239, see Table 5). The *pfcrt* c76T polymorphism and the *pfcrt* c72–76 CVIET haplotype, were found in 52.4% and 43.5%, respectively. Almost all of the samples (90%) were *pfmdr1* c86N. The *pfdhfr* triple mutant was present at 82.4%. The *pfdhps* c437G polymorphism was 66.8%, while the polymorphisms at codons 540, 581 and 613 known to occur further downstream in *pfdhps* in an East African context were either absent or only found in single samples. The *pfdhps* c431V, which recently has been found in Nigeria and has been proposed as a molecular reason for the lack of mutations downstream of c437 in *pfdhps* in West-African *P. falciparum* parasites^49, 50^ was only found in one sample. These findings are consistent with previously published findings from the same area^51^, while also indicating an increase in the frequency of *pfmdr1* c86N^51^, consistent with the recommendation and use of both AL and QN in treatment of malaria in Guinea-Bissau^1, 12, 52^. Furthermore, the frequency of the *pfdhfr/pfdhps* quadruple mutant was 52.8%, which indicates a noteworthy increase since it was last documented in 2004 at 15%^53^. Selection of the *pfdhfr/pfdhps* quadruple mutant may be explained by the recommendation and use of SP for IPTp in Guinea-Bissau, as well as through possible import from neighbouring countries with higher levels of the quadruple mutant^49, 52, 54, 55^.

## Conclusion

The methodology presented in this study represents an affordable, high throughput and reliable platform for efficient surveillance of spread and emergence of antimalarial drug resistance. With Illumina technology already installed in several research facilities in countries with malaria, implementing surveillance of antimalarial drug resistance based on this method would be feasible in these locations. In the current study, the analysis of all molecular markers of drug resistance was performed for less than 10 USD per sample, but can be run for as little as 4 USD per sample. Between 3 and 16 amplicons from 457 *P. falciparum* patient samples were successfully sequenced on a single Miseq v3 flow cell with an average coverage of 2,043 reads and a minimum z-score of 1.96 applying to all published base calls.

## Methods

### Patients and malaria parasite samples

A demographic overview of the patients recruited and the sampling process is depicted in Fig. 1. Patients of all ages attending the Bandim and Belem health care centres in Guinea-Bissau between October 2014 and March 2016, symptomatic of and diagnosed with *P. falciparum* malaria using malaria RDTs were after informed consent asked to donate blood for filter paper sampling (n = 318). Blood samples were drawn in accordance with the relevant guidelines and regulations. An additional 139 patients donated their *P. falciparum* positive RDTs. All patients donating samples had fever (≥37, 5 °C), or a history of fever within the past 24 hours and hence qualified as having clinical malaria and were treated according to national malaria treatment guidelines. Filter paper samples were collected as venous blood, which was filtered with Plasmodipur filters (Europroxima, Arnhem, Netherlands), and dotted on Whatman filter paper nr. 3. Filter paper and RDTs were stored at room temperature for 6–9 months before transport to Denmark. Furthermore, 6 well-described parasite isolates (3D7, Dd2, K1, 7G8, MRA-1238, and MRA-1239) were used as controls. The data acquired for these control strains is listed in Table 5.

### Ethical approval

Ethical approval and permission for sampling venous blood from malaria patients in Guinea-Bissau was given by the ethical review board in Bissau (ref: 022/CNES/INASA/2014, dated September 17th 2014). Filter paper samples were acquired after written informed consent from the patient or their guardian. Blood samples were drawn in accordance with the relevant guidelines and regulations.

### DNA extraction

DNA was extracted according to the chelex method described elsewhere^56^.

### Primer design

The experimental protocol was designed to investigate polymorphisms occurring in *pfcrt* (c72–76), *pfmdr1* (c86, c1034, c1042 and c1246), *pfdhfr* (c16, c51, c59, c108 and c164), *pfdhps* (c431, c436, c437, c540, c581 and c613), all of *pfK13*, and mitochondrial-genome base positions (772, 853, 973, 1283 and 2383). All gene-accession numbers, PCR fragments and SNPs are illustrated in Fig. 3. The entire protocol for PCRs and Miseq sequencing is illustrated in Figure S1. Primers were designed based on the principles described in Illumina’s own Miseq protocol for 16 S metagenomic sequencing library preparation^57^ and are listed in Supplementary Tables S1 (gene specific primers) and S2 (index primers). The concept of gene-specific and index PCR as well as characteristics of the primers needed for each step are illustrated in Fig. 2. The gene-specific primers were designed to anneal to the *P. falciparum* gene of interest (at a melting temperature around 60 °C), amplifying a fragment of approximately 500–550 bp, in order to allow for overlap of the two reads conducted during paired-end sequencing (350 bp reads). The gene-specific primers all contain a non-annealing overhang on both forward and reverse primers. The overhangs are incorporated into both ends of the PCR product during the gene-specific PCR, and can now act as annealing site for the indexing primers (Table S2) during the indexing PCR. The overhang sequence is based on Illumina’s protocol, because it also serves as primer annealing site for Illumina’s own sequencing primers during sequencing. The length and melting temperature are therefore ideal for the temperatures applied by the Miseq during the different sequencing steps. Indexing primers were designed to anneal to the overhangs, while themselves containing overhangs consisting of individual 8-base indices and adapter sequences that will allow the final PCR product to bind to the sequencing flow cell. Indices were generated using the Barcode Generator V. 2.8^58^, indicating a desired barcode length of 8 bases, a distance between barcodes of 4 bases and a GC content of at least 40%. All indices used for generation of the presented data are listed in Table S3. All primers were ordered at Eurofins Genomics (MWG-Biotech AG, Edersberg, Germany).

### PCR reactions and programs

All PCRs were performed with AH PCR Mastermix (AH diagnostics, Århus, Denmark). Four different multiplex PCR reactions were run, while Pfcrt.2 and Mito.2 were run as simplex. The multiplex primer combinations are listed in Table S1. Gene-specific primers were used at a 0,065 μM final concentration each, together with 1 μl of template DNA in a total reaction volume of 10 μl. The gene-specific PCR program consisted of the following steps: Heat activation at 95 °C for 15 minutes, 30 cycles of denaturation at 95 °C for 20 seconds, annealing at 57 °C for 2 minutes, and elongation at 72 °C for 2 minutes, and one final elongation at 72 °C for 10 minutes. In order to target relatively uniform coverage of the various amplicons, a subset of the PCR products from the index-specific multiplex PCRs were analysed by qPCR for the relative presence of the different fragments (qPCR described below). Based on this data (not presented) the PCR products were mixed at a ratio of (multiplex PCRs 1–4) M1:M2:M3:M4/1:0,5:4:6. M5 and amplicon Mito.2 were mixed at a ratio of 2:1, and Pfcrt.2 was run as simplex on the index PCR. These mixtures of multiplexes were used as template in the indexing PCRs. Indexing PCR reactions were performed with a final primer concentration of 0,065 μM together with 0,75 μl of gene-specific PCR product (mix of multiplexes or simplex reactions) in a final volume of 11,75 μl. The indexing PCR program consisted of the following steps: Heat activation at 95 °C for 15 minutes, 20 cycles of denaturation at 95 °C for 20 seconds, annealing at 60 °C for 1 minute, and elongation at 72 °C for 1 minute, and one final elongation at 72 °C for 10 minutes. Reactions were performed on either a VWR DuoCycler, VWR UNO96 (VWR, Søborg, Denmark) or an MWG BIOTECH Primus96 Plus (MWG-BIOTECH, Edersberg, Germany).

### qPCR

Primers applied for qPCR analysis are listed in Table S3. qPCR reactions were performed with QuantiTect SYBR Green Master Mix (Qiagen, Hilden, Germany), a final primer concentration of 2 μM in a final volume of 20 μl. Template for the reaction consisted of 1 μl of PCR product obtained from the Index PCR, diluted 1:200. The qPCR program consisted of the following steps: Heat activation at 95 °C for 15 minutes, 20 cycles of denaturation at 95 °C for 30 seconds, annealing at 54 °C for 40 seconds, and elongation at 68 °C for 50 seconds, and one final elongation at 68 °C for 10 minutes. See Figure S2 for example data.

### Amplicon purification, dilution and pooling

Pools were made for a maximum of 96 samples, consisting of 4 ul of each indexing PCR reaction, totalling 14 pools (corresponding to the 14 individual 96-well plates that the PCRs were run in). The amplicons were bead purified with AMPure XP beads (Beckman Coulter, California, United States) according to manufacturer’s protocol, using 200 ul PCR product (per pool), and 0.6 x PCR-pool volume of beads (=120 ul), in order to eliminate primer dimers of approximately 250 bp. Purified PCR pools were analysed on Agilent 2100 Bioanalyser using the Bioanalyser 1000 Kit (Agilent Technologies, Santa Clara, CA, USA), to verify the proper elimination of primer dimers, and amplicon sizes of approximately 650–700 bp. Concentration of purified PCR pools were measured on a Nanodrop2000 UV-VIS Spectrophotometer (Thermo Fisher Scientific, Waltham, MA, USA) and subsequently diluted to 4 nM pools, according to the following equation: (concentration of pool/(660 * fragment length (bp))) * 1,000,000. Finally, diluted amplicon pools were pooled further according to the space each pool was to be given on the flow cell during sequencing (described in Figure S3). This was decided based on the amount of samples represented by each pool, multiplied by the amount of fragments amplified for each sample in the pool.

### Miseq sequencing

The pooled libraries were denatured with NaOH to a final concentration of 1 mM, diluted with hybridization buffer and further heat denatured at 96 °C for 2 min before running the sequencing. 5% PhiX was included for low diversity libraries. The sequencing was performed with an Illumina MiSeq instrument using paired end 2 × 300-bp reads and a MiSeq v3 flow cell according to the manufacturers recommendation. Sequencing was carried out by the DTU in-house facility (DTU Multi-Assay Core (DMAC), Technical University of Denmark).

### Quality trimming of paired-end sequences and base calling

Raw sequences were quality trimmed with *Cutadapt* ^59^ at a phred score of 20, and primer sequences were trimmed from the 5′-end of the sequences, in order to avoid primer bias in the sequenced fragments. Base calling was performed with *Assimpler* ^60^, a *Python* program developed to compare reads with a custom database, consisting of the 3D7 reference sequences of all the analysed genes. *Assimpler* will then output two files, a column file with the reference base and the called base from the sample at all positions in the custom database, as well as a contigs file. Bases were called with a minimum z-score of 1.96 corresponding to a p-value below 0,05. The Z-score was calculated as Z = (X − Y)/sqrt(X + Y), the number of reads X having the most common nucleotide at that position, and the number of reads Y supporting other nucleotides^60^. Mixed infections were categorised as samples with <75% unanimous reads for a given base position for the purpose of generating haplotype data.

## Electronic supplementary material

Supplementary information

## References

[1] WHO. *World Malaria Report 2015*, (May 29th 2016) http://apps.who.int/iris/bitstream/10665/200018/1/9789241565158_eng.pdf (2016).

[2] Dondorp AM (2010). Artemisinin resistance: current status and scenarios for containment. Nature reviews. Microbiology.

[3] Menard D (2016). A Worldwide Map of Plasmodium falciparum K13-Propeller Polymorphisms. The New England journal of medicine.

[4] Meshnick SR, Taylor TE, Kamchonwongpaisan S (1996). Artemisinin and the antimalarial endoperoxides: from herbal remedy to targeted chemotherapy. Microbiological reviews.

[5] Cowman AF, Morry MJ, Biggs BA, Cross GA, Foote SJ (1988). Amino acid changes linked to pyrimethamine resistance in the dihydrofolate reductase-thymidylate synthase gene of Plasmodium falciparum. Proceedings of the National Academy of Sciences of the United States of America.

[6] Peterson DS, Walliker D, Wellems TE (1988). Evidence that a point mutation in dihydrofolate reductase-thymidylate synthase confers resistance to pyrimethamine in falciparum malaria. Proceedings of the National Academy of Sciences of the United States of America.

[7] Triglia T, Menting JG, Wilson C, Cowman AF (1997). Mutations in dihydropteroate synthase are responsible for sulfone and sulfonamide resistance in Plasmodium falciparum. Proceedings of the National Academy of Sciences of the United States of America.

[8] Fidock DA (2000). Mutations in the P. falciparum digestive vacuole transmembrane protein PfCRT and evidence for their role in chloroquine resistance. Molecular cell.

[9] Reed MB, Saliba KJ, Caruana SR, Kirk K, Cowman AF (2000). Pgh1 modulates sensitivity and resistance to multiple antimalarials in Plasmodium falciparum. Nature.

[10] Djimde A (2001). A molecular marker for chloroquine-resistant falciparum malaria. The New England journal of medicine.

[11] Sidhu AB, Valderramos SG, Fidock D (2005). A. pfmdr1 mutations contribute to quinine resistance and enhance mefloquine and artemisinin sensitivity in Plasmodium falciparum. Molecular microbiology.

[12] Mwai L (2009). *In vitro* activities of piperaquine, lumefantrine, and dihydroartemisinin in Kenyan Plasmodium falciparum isolates and polymorphisms in pfcrt and pfmdr1. Antimicrobial agents and chemotherapy.

[13] Dondorp AM (2009). Artemisinin resistance in Plasmodium falciparum malaria. The New England journal of medicine.

[14] Phyo AP (2012). Emergence of artemisinin-resistant malaria on the western border of Thailand: a longitudinal study. Lancet.

[15] Ashley EA (2014). Spread of artemisinin resistance in Plasmodium falciparum malaria. The New England journal of medicine.

[16] Organization, W. H. Status Report on Artemisinin Resistance (2014).

[17] Ariey F (2014). A molecular marker of artemisinin-resistant Plasmodium falciparum malaria. Nature.

[18] Veiga MI (2016). Globally prevalent PfMDR1 mutations modulate Plasmodium falciparum susceptibility to artemisinin-based combination therapies. Nature communications.

[19] Eastman RT, Khine P, Huang R, Thomas CJ, Su XZ (2016). PfCRT and PfMDR1 modulate interactions of artemisinin derivatives and ion channel blockers. Scientific reports.

[20] Sutherland CJ (2009). Novel pfdhps haplotypes among imported cases of Plasmodium falciparum malaria in the United Kingdom. Antimicrobial agents and chemotherapy.

[21] Takala-Harrison S (2014). Independent Emergence of Artemisinin Resistance Mutations Among Plasmodium falciparum in Southeast Asia. The Journal of infectious diseases.

[22] Organization, W. H. *WHO Evidence Review Group on Intermittent Preventive Treatment (IPT) of malaria in pregnancy*. (May 29th 2016) Globally prevalent PfMDR1 mutations modulate Plasmodium falciparum susceptibility to artemisinin-based combination therapies (2013). 10.1038/ncomms11553 PMC4873939 27189525

[23] Taylor, S. M. *et al*. Absence of Putative Artemisinin Resistance Mutations Among Plasmodium falciparum in Sub-Saharan Africa: A Molecular Epidemiologic Study. *The Journal of infectious diseases*, doi:10.1093/infdis/jiu467 (2014). 10.1093/infdis/jiu467 PMC4402372 25180240

[24] Kamau E (2015). K13-propeller polymorphisms in Plasmodium falciparum parasites from sub-Saharan Africa. The Journal of infectious diseases.

[25] Malaria, G. E. N. P. f. C. P. Genomic epidemiology of artemisinin resistant malaria. *eLife* **5**, doi:10.7554/eLife.08714 (2016). 10.7554/eLife.08714 PMC4786412 26943619

[26] Preston MD (2014). A barcode of organellar genome polymorphisms identifies the geographic origin of Plasmodium falciparum strains. Nature communications.

[27] Duraisingh MT, Curtis J, Warhurst DC (1998). Plasmodium falciparum: detection of polymorphisms in the dihydrofolate reductase and dihydropteroate synthetase genes by PCR and restriction digestion. Experimental parasitology.

[28] Durand R (2000). Use of molecular beacons to detect an antifolate resistance-associated mutation in Plasmodium falciparum. Antimicrobial agents and chemotherapy.

[29] Ranford-Cartwright LC, Johnston KL, Abdel-Muhsin AM, Khan BK, Babiker HA (2002). Critical comparison of molecular genotyping methods for detection of drug-resistant Plasmodium falciparum. Transactions of the Royal Society of Tropical Medicine and Hygiene.

[30] Nair S, Brockman A, Paiphun L, Nosten F, Anderson TJ (2002). Rapid genotyping of loci involved in antifolate drug resistance in Plasmodium falciparum by primer extension. International journal for parasitology.

[31] Wilson PE, Alker AP, Meshnick SR (2005). Real-time PCR methods for monitoring antimalarial drug resistance. Trends in parasitology.

[32] Alifrangis M (2005). A simple, high-throughput method to detect Plasmodium falciparum single nucleotide polymorphisms in the dihydrofolate reductase, dihydropteroate synthase, and P. falciparum chloroquine resistance transporter genes using polymerase chain reaction- and enzyme-linked immunosorbent assay-based technology. The American journal of tropical medicine and hygiene.

[33] Cruz RE, Shokoples SE, Manage DP, Yanow SK (2010). High-throughput genotyping of single nucleotide polymorphisms in the Plasmodium falciparum dhfr gene by asymmetric PCR and melt-curve analysis. Journal of clinical microbiology.

[34] Campino S (2011). Population genetic analysis of Plasmodium falciparum parasites using a customized Illumina GoldenGate genotyping assay. PloS one.

[35] Kamau E (2012). Development of a TaqMan Allelic Discrimination assay for detection of single nucleotides polymorphisms associated with anti-malarial drug resistance. Malaria journal.

[36] Daniels R (2012). Rapid, field-deployable method for genotyping and discovery of single-nucleotide polymorphisms associated with drug resistance in Plasmodium falciparum. Antimicrobial agents and chemotherapy.

[37] Nankoberanyi S (2014). Validation of the ligase detection reaction fluorescent microsphere assay for the detection of Plasmodium falciparum resistance mediating polymorphisms in Uganda. Malaria journal.

[38] Aydin-Schmidt B (2014). Loop mediated isothermal amplification (LAMP) accurately detects malaria DNA from filter paper blood samples of low density parasitaemias. PloS one.

[39] Jacob CG (2014). A microarray platform and novel SNP calling algorithm to evaluate Plasmodium falciparum field samples of low DNA quantity. BMC genomics.

[40] Taylor SM (2013). Pooled deep sequencing of Plasmodium falciparum isolates: an efficient and scalable tool to quantify prevailing malaria drug-resistance genotypes. The Journal of infectious diseases.

[41] Rao PN (2016). A Method for Amplicon Deep Sequencing of Drug Resistance Genes in Plasmodium falciparum Clinical Isolates from India. Journal of clinical microbiology.

[42] Amato, R. *et al*. Genetic markers associated with dihydroartemisinin-piperaquine failure in Plasmodium falciparum malaria in Cambodia: a genotype-phenotype association study. *The Lancet. Infectious diseases*, doi:10.1016/S1473-3099(16)30409-1 (2016). 10.1016/S1473-3099(16)30409-1 PMC5564489 27818095

[43] Schirmer M (2015). Insight into biases and sequencing errors for amplicon sequencing with the Illumina MiSeq platform. Nucleic acids research.

[44] Conrad MD (2014). Polymorphisms in K13 and falcipain-2 associated with artemisinin resistance are not prevalent in Plasmodium falciparum isolated from Ugandan children. PloS one.

[45] Torrentino-Madamet M (2014). Limited polymorphisms in k13 gene in Plasmodium falciparum isolates from Dakar, Senegal in 2012-2013. Malaria journal.

[46] Kamau E (2014). K13-Propeller Polymorphisms in Plasmodium falciparum Parasites From Sub-Saharan Africa. The Journal of infectious diseases.

[47] Beshir KB (2013). Residual Plasmodium falciparum parasitemia in Kenyan children after artemisinin-combination therapy is associated with increased transmission to mosquitoes and parasite recurrence. The Journal of infectious diseases.

[48] Muwanguzi J (2016). Lack of K13 mutations in Plasmodium falciparum persisting after artemisinin combination therapy treatment of Kenyan children. Malaria journal.

[49] Chauvin P (2015). Prevalence of Plasmodium falciparum parasites resistant to sulfadoxine/pyrimethamine in pregnant women in Yaounde, Cameroon: emergence of highly resistant pfdhfr/pfdhps alleles. The Journal of antimicrobial chemotherapy.

[50] Oguike MC (2016). Molecular determinants of sulfadoxine-pyrimethamine resistance in Plasmodium falciparum in Nigeria and the regional emergence of dhps 431V. *International journal for parasitology*. Drugs and drug resistance.

[51] Jovel IT, Kofoed PE, Rombo L, Rodrigues A, Ursing J (2015). Temporal and seasonal changes of genetic polymorphisms associated with altered drug susceptibility to chloroquine, lumefantrine, and quinine in Guinea-Bissau between 2003 and 2012. Antimicrobial agents and chemotherapy.

[52] Cheruiyot J (2014). Polymorphisms in Pfmdr1, Pfcrt, and Pfnhe1 genes are associated with reduced *in vitro* activities of quinine in Plasmodium falciparum isolates from western Kenya. Antimicrobial agents and chemotherapy.

[53] Kofoed PE (2004). Genetic markers of resistance to pyrimethamine and sulfonamides in Plasmodium falciparum parasites compared with the resistance patterns in isolates of Escherichia coli from the same children in Guinea-Bissau. Tropical medicine & international health: TM & IH.

[54] Ndiaye D (2013). Polymorphism in dhfr/dhps genes, parasite density and *ex vivo* response to pyrimethamine in Plasmodium falciparum malaria parasites in Thies, Senegal. *International journal for parasitology*. Drugs and drug resistance.

[55] Coulibaly SO (2014). Parasite clearance following treatment with sulphadoxine-pyrimethamine for intermittent preventive treatment in Burkina-Faso and Mali: 42-day *in vivo* follow-up study. Malaria journal.

[56] Schriefer ME, Sacci JB, Wirtz RA, Azad AF (1991). Detection of polymerase chain reaction-amplified malarial DNA in infected blood and individual mosquitoes. Experimental parasitology.

[57] Illumina. *16S Metagenomic Sequencing Library Preparation - Preparing 16S Ribosomal RNA Gene Amplicons for the Illumina MiSeq System*. (May 4th 2016) http://www.illumina.com/content/dam/illumina-support/documents/documentation/chemistry_documentation/16s/16s-metagenomic-library-prep-guide-15044223-b.pdf (2013).

[58] Comai, L. & Howell, T. *Barcode Generator v. 2.8*, (May 4th 2016) http://comailab.genomecenter.ucdavis.edu/index.php/Barcode_generator (2012).

[59] Martin M (2011). Cutadapt Removes Adapter Sequences From High-Throughput Sequencing Reads. EMBnet. journal.

[60] Leekitcharoenphon P, Nielsen EM, Kaas RS, Lund O, Aarestrup FM (2014). Evaluation of whole genome sequencing for outbreak detection of Salmonella enterica. PloS one.

