# Supplementary material for: High throughput resistance profiling of Plasmodium falciparum infections based on custom dual indexing and Illumina next generation sequencing-technology
Source: Sci Rep. 2017 May 25;7:2398. doi: 10.1038/s41598-017-02724-x (PMC5445084; doi:10.1038/s41598-017-02724-x)
Supplement: Supplementary file 1 — Supplementary information [file 41598_2017_2724_MOESM1_ESM.pdf]

High throughput resistance profiling of *Plasmodium falciparum* infections based on custom dual indexing and Illumina next generation sequencing-technology

Sidsel Nag[1,2]*, Marlene D. Dalgaard[3], Poul-Erik Kofoed[4,5], Johan Ursing[5,6], Marina Crespo[1,2], Lee O'Brien Andersen[7], Frank Møller Aarestrup[8], Ole Lund[3] and Michael Alifrangis[1,2].

Legends for supplementary figures 1-3

Figure S1. Protocol overview

Library preparation for the sequencing performed in the method described in this study, is entirely PCR-based. Gene-specific fragments are amplified from the *P. falciparum* specimens in the patient samples, through a number of multiplex PCRs ("gene-specific PCR" see Figure 2 for detailed description), followed by a PCR where the individual index combinations are incorporated into the PCR products ("Index PCR", see Figure 2 for detailed description). At this point, PCR positivity and extent of primer dimer formation are checked on agarose gels. Furthermore, qPCR is applied to assess the relative presence of the various gene-fragments, in order to potentially optimise ratios of the various gene-fragments obtained (see Figure S2 for example data). Samples are now combined into pools, in order to minimise material use and work load during bead purification. The pools are then purified with DNA-binding beads, and the elutions are all checked with a bioanalyser for size-distributions of their content. Excessive presence of primer dimer will also be visible at this point. The concentrations of the purified pools can now be measured and dilutions can be made accordingly. Once equimolar dilutions have been made, these can be combined into a sequencing library, where the amount added of each diluted pool corresponds to the space devoted to the content of this pool on the flow cell (see Figure S3 for example data). Finally, the library can be applied for sequencing.

Figure S2. Adjustment of gene-specific PCR product applied in index PCR, analyzed by qPCR

Relative product difference obtained after running the index PCR was analyzed using qPCR, as described in methods, applying the primers listed in Table S3 (for details on which products are amplified together in multiplex PCR reactions, see Table S1). The qPCR data shown are average data for 10 analyzed samples. The relative product difference was found by using the following equations:

1) Δ CT = CT Amplicon – CT PfK13.1
2) Relative product difference = 2^(- Δ CT)

Figure S2a shows the relative product difference when input from M1:M2:M3:M4 was 1:1:1:1 and M5:Mito.2 were also 1:1. Pfcrt.2 was run as simplex in the index PCR. Figure S2b shows the relative product difference when input from M1:M2:M3:M4 was 1:0,5:4:6 and M5:Mito.2 were 2:1. Pfcrt2 was run as simplex.

Figure S3: Combining diluted amplicon pools in final sequencing library

The various pools created during step 4 in Figure S1, have to be combined into a single sequencing library prior to sequencing (step 8 in Figure S1). The pools may contain a varying amount of samples and even a varying amount of amplicons representing each sample. In the current study, pools were created simply according to individual 96-well PCR plates. Therefore some pools contained samples corresponding to a full plate, and others much fewer. Furthermore, due to the multiplexing in the index PCR, some PCR plates contained 11 amplicons representing each sample, while others only contained 1 or 2. If these pools were combined as equal amounts from each pool, the pools containing the fewest samples and amplicons (combined denoted as units in the figure) would acquire a much deeper sequencing than others with more units. Therefore differentiated pooling should be performed to normalise sequencing depth as much as possible. Alternatively, select samples can also be sequenced deeper than others, by applying the opposite approach.

Legends for supplementary tables 1-3

Table S1: Gene-specific primers

The table lists all of the primers applied to amplify the *P. falciparum* gene fragments sequenced in this study, as well as the multiplex PCR reaction in which the individual primer sets were incorporated. See Figure 3 for the location of the various fragments within the genes.

Table S2: Index primers

The table lists all of the index primers generated for this study, allowing for 2450 unique index combinations. Index primers were never combined with their respective reverse-complement partner (i.e. Index_1_F was never combined with Index_1_R). Combining reverse-complement partners increases the risk of primer dimers. The total number of unique combinations therefore amounts to 50 x 50 – 50 = 2450 combinations.

Table S3: qPCR primers

The table lists all of the qPCR primers applied to validate the relative amplification of the gene fragments in the various multiplex PCRs, when select samples were checked for quality and content (see Figure S1 for protocol overview).

Image 1/2

1. Gene-specific PCR
Gene-specific primers encoding identical overhangs are used to amplify parasite genes of interest, and incorporating the overhangs into the resulting PCR products (see Figure 2 for details and Table S1 for gene-specific primers).

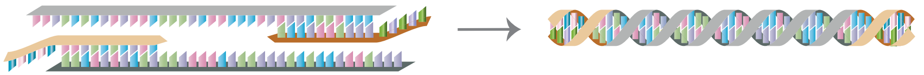

2. Index PCR
PCR products from step 1 are used as template together with index primers, which anneal to the overhangs. Individual index combinations are now incorporated into the final PCR product (from here on referred to as amplicons, see Figure 2 for details and Table S2 for index primers).

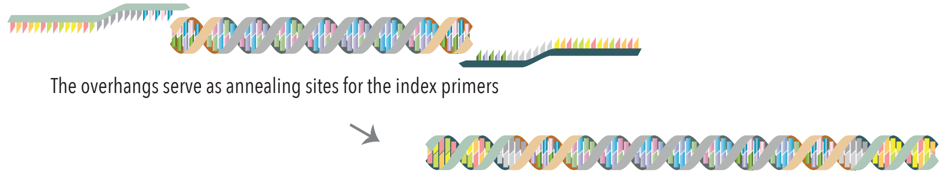

3. Quality check of select samples
Agarose gels and qPCR can be performed on select samples from step 2, to verify the presence of the wanted amplicons, absence of primer dimers and relative abundance of various fragments amplified in individual multiplex PCR reactions performed in step 1 and 2 (see Table S3 for qPCR primers)

If visualisation on agarose gels indicates absence of the wanted amplicons (approx. 750 bp) and primer dimers are overwhelming (approx. 250 bp), it is recommended to check for presence of PCR products in step 1, and then repeat steps 1 and/or 2, potentially optimising PCR-reaction settings (ladder depicted is 100 bp). Furthermore, if qPCR results indicate major differences in the abundance of the different fragments after performing step 2, it is recommended to repeat step 2, changing the ratios of the input from step 1 (see Figure S2 for data example from qPCR quality check).

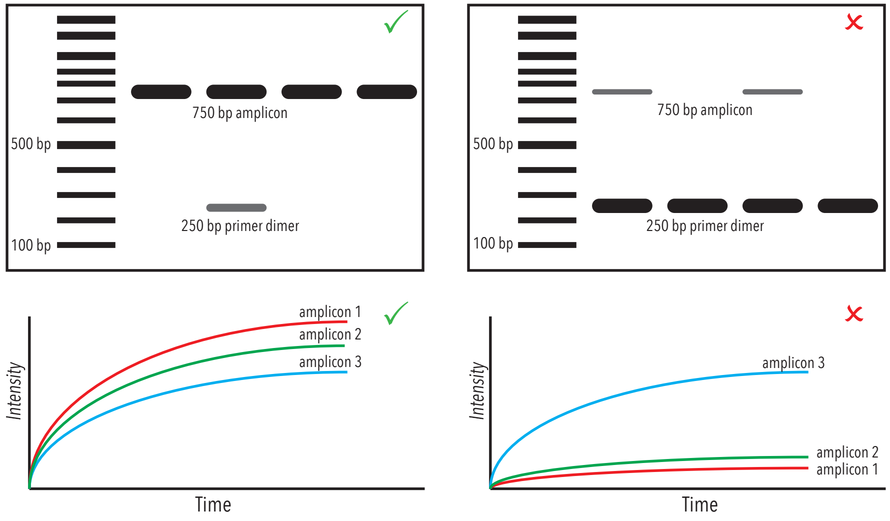

4. Sample pooling
Equal amounts of all PCR reactions are mixed in pools to increase the sample volume and minimize sample quantity for downstream protocol steps. Pooling of PCR reactions containing the same amplicons is recommended for simplification of the bioanalyzer control in step 6.

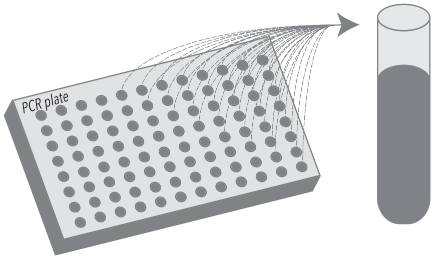

5. Bead purification of PCR pools

DNA-binding beads are mixed with the pooled amplicons, in order to purify the amplicons from PCR reaction-reagents. The bead:PCR product ratio (0.6:1) is chosen for optimal separation of the 750 bp amplicons from the 250 bp primer dimers.

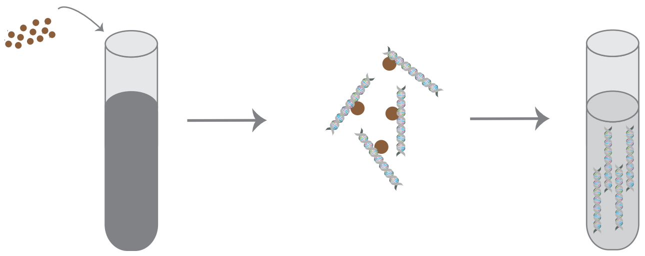

6. Quality Check of purified PCR products

Eluates containing the purified amplicons are analyzed on a Bioanalyzer, to assess sample purity. If samples are heavily contaminated with primer dimer, it is recommended to repeat step 5. The "lower marker" and "higher marker" depicted represent the standard markers contained in the Bioanalyzer HS kit.

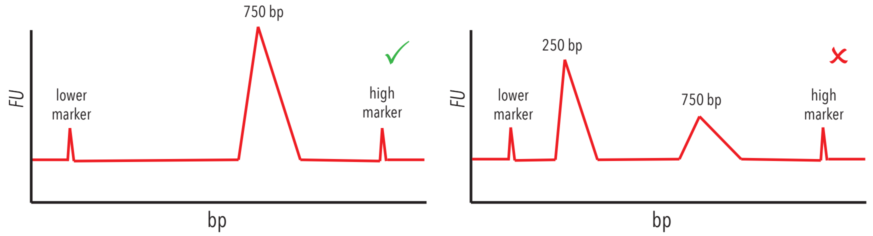

7. Dilution of purified pools

Concentration measurements are now performed on the purified pools of amplicons. These are then diluted to 4 nM, according to the equation: (concentration of pool/(660*fragment length (bp)))*1,000,000.

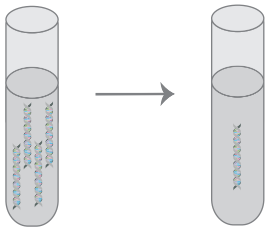

8. Combination of pools in amplicon library

A small volume from each 4nM pool can now be combined in a single amplicon library. If certain pools are to be given more space than others on the flow cell (for deeper sequencing or because those pools contain more samples), the amount added from these pools to the library, is adjusted accordingly (see Figure S3 for data example).

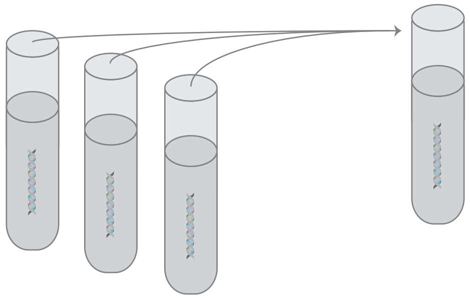

9. Sequencing

The amplicon library prepared in step 8 is applied according to Illumina's Nextera protocol for sequencing on the Illumina Miseq, applying a V3 flow cell.

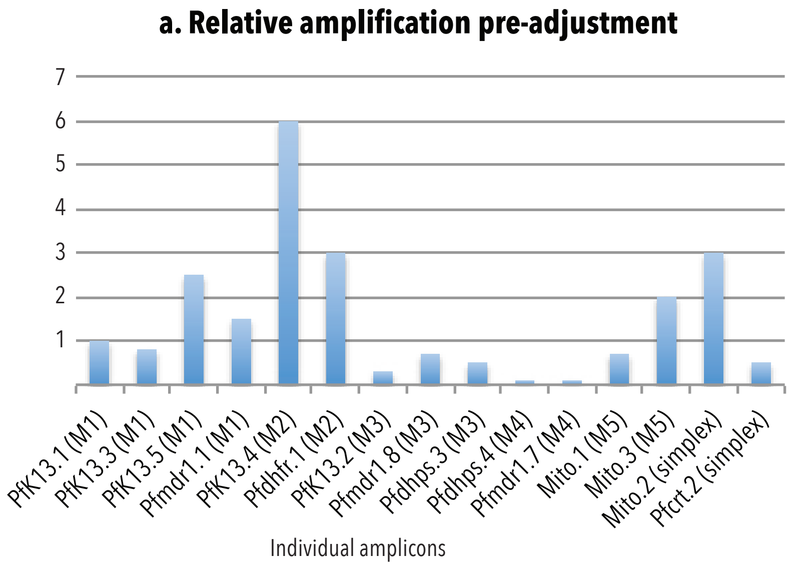

a. Relative amplification pre-adjustment
7
6
5
4
3
2
1
PfK13.1 (M1)
PfK13.3 (M1)
PfK13.5 (M1)
Pfmdr1.1 (M1)
PfK13.4 (M2)
Pfdhfr.1 (M2)
PfK13.2 (M3)
Pfmdr1.8 (M3)
Pfdhps.3 (M3)
Pfdhps.4 (M4)
Pfmdr1.7 (M4)
Mito.1 (M5)
Mito.3 (M5)
Mito.2 (simplex)
Pfcrt.2 (simplex)
Individual amplicons
b. Relative amplification post-adjustment
7
6
5
4
3
2
1
PfK13.1 (M1)
PfK13.3 (M1)
PfK13.5 (M1)
Pfmdr1.1 (M1)
PfK13.4 (M2)
Pfdhfr.1 (M2)
PfK13.2 (M3)
Pfmdr1.8 (M3)
Pfdhps.3 (M3)
Pfdhps.4 (M4)
Pfmdr1.7 (M4)
Mito.1 (M5)
Mito.3 (M5)
Mito.2 (simplex)
Pfcrt.2 (simplex)
Individual amplicons

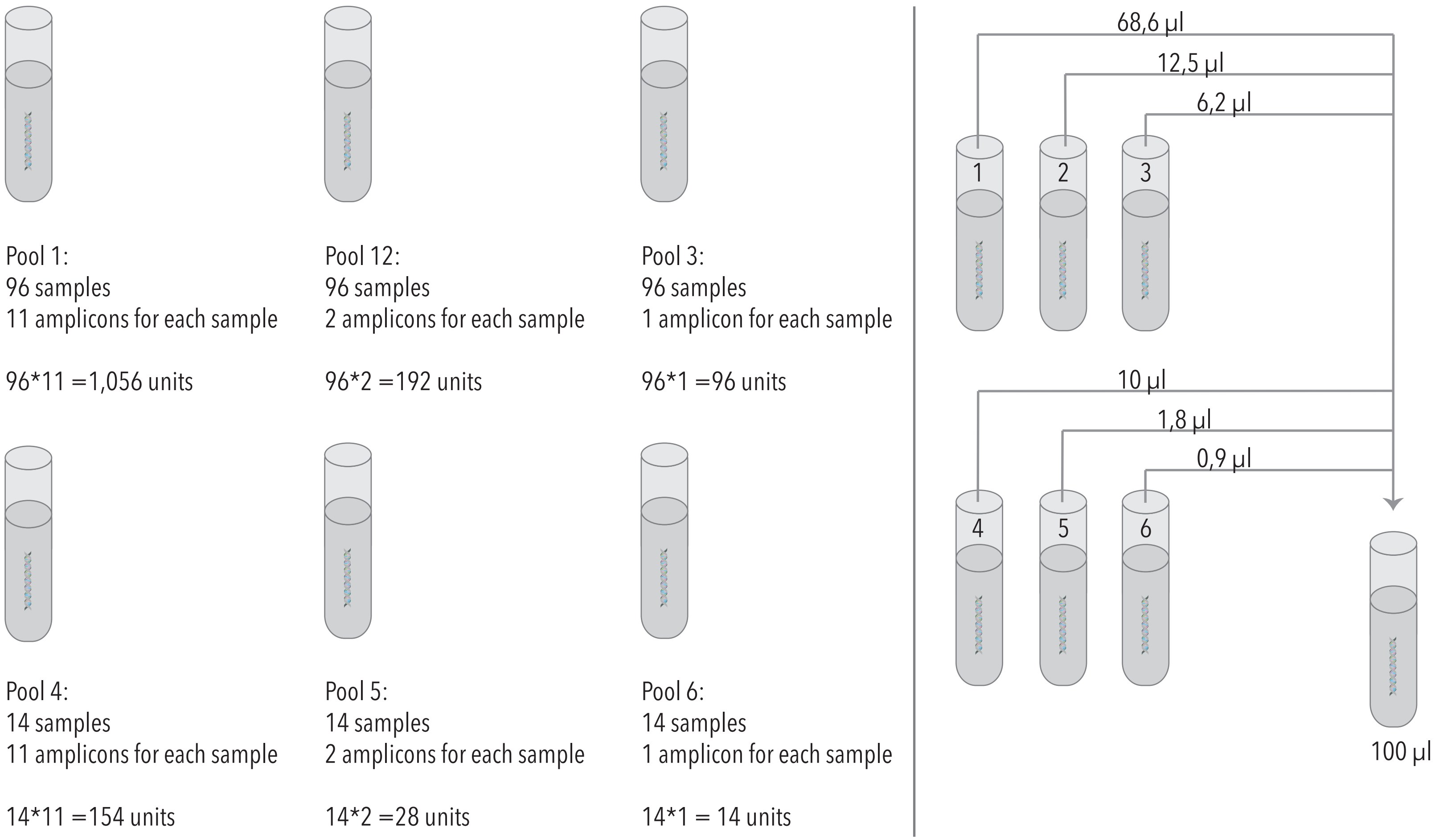

Combined, the pools yeild 1540 units.When combining the pools, in a final sequencing library resulting in a final volume of 100 µl, the amounts added from each pool are found according to the following equation:

Added amount from individual pool = total number of units within individual pool / total number of units in pools combined * final volume of library
Example for pool 1:
1,056 units / 1540 units *100 µl = 68,6 µl

Table S1. Gene-specific primers and multiplex PCR reaction combinations

Fragment name	Ref. Gene		Primer sequence	PCR reaction
Pfdhfr.1	PF3D7_0417200	Fw.	TCGTCGGCAGCGTCAGATGTGTATAAGAGACAG**ATGATGGAACAAGTCTGCGACGTTTTCGA**	**Multiplex 2 (M2)**
		Rev.	GTCTCGTGGGCTCGGAGATGTGTATAAGAGACAG**CTAAAAATTCTTGATAAACAACGGAACCTCC**	
Pfmdr1.1	PF3D7_0523000	Fw.	TCGTCGGCAGCGTCAGATGTGTATAAGAGACAG**ATGGGTAAAGAGCAGAAAGAGAAAAAAGATG**	**Multiplex 1 (M1)**
		Rev.	GTCTCGTGGGCTCGGAGATGTGTATAAGAGACAG**GTTATAAATTTCGTACCAATTCCTGAACTCA**	
Pfmdr1.7		Fw.	TCGTCGGCAGCGTCAGATGTGTATAAGAGACAG**TTTGTCCAATTGTTGCAGCTGTATTAACTTT**	**Multiplex 4 (M4)**
		Rev.	GTCTCGTGGGCTCGGAGATGTGTATAAGAGACAG**TGCATTTTCTGAATCTCCTTTTAAGGACATT**	
Pfmdr1.8		Fw.	TCGTCGGCAGCGTCAGATGTGTATAAGAGACAG**GGTAAAGTTGATATTAAAGATGTAAATTTCC**	**Multiplex 3 (M3)**
		Rev.	GTCTCGTGGGCTCGGAGATGTGTATAAGAGACAG**TGGTCCAACATTTGTATCATATTTATTTGG**	
Pfcrt.2	PF3D7_0709000	Fw.	TCGTCGGCAGCGTCAGATGTGTATAAGAGACAG**TGGCTCACGTTTAGGTGGAGGTTCTTG**	Simplex
		Rev.	GTCTCGTGGGCTCGGAGATGTGTATAAGAGACAG**ACTGAACAGGCATCTAACATGGATATAGC**	
Pfdhps.3	PF3D7_0810800	Fw.	TCGTCGGCAGCGTCAGATGTGTATAAGAGACAG**ACCATCAGATGTTTATATAACAAATATGTG**	**Multiplex 3 (M3)**
		Rev.	GTCTCGTGGGCTCGGAGATGTGTATAAGAGACAG**CTGGATTATTTGTACAAGCACTAATATCA**	
Pfdhps.4		Fw.	TCGTCGGCAGCGTCAGATGTGTATAAGAGACAG**AGAATGTGTTGATAATGATTTAGTTGATAT**	**Multiplex 4 (M4)**
		Rev.	GTCTCGTGGGCTCGGAGATGTGTATAAGAGACAG**GATATAAAAGTTGATCCTTGTCTTTCCT**	
PfK13.1	PF3D7_1343700	Fw.	TCGTCGGCAGCGTCAGATGTGTATAAGAGACAG**ATGGAAGGAGAAAAAGTAAAAACAAAAGC**	**Multiplex 1 (M1)**
		Rev.	GTCTCGTGGGCTCGGAGATGTGTATAAGAGACAG**GTTGGTATTCATAATTGATGGAGAATTC**	
PfK13.2		Fw.	TCGTCGGCAGCGTCAGATGTGTATAAGAGACAG**CTGACAGCAAATAATATAACTAATAATCT**	**Multiplex 3 (M3)**
		Rev.	GTCTCGTGGGCTCGGAGATGTGTATAAGAGACAG**TCTTCATCAAATCGTTTCCTATGTT**	
PfK13.3		Fw.	TCGTCGGCAGCGTCAGATGTGTATAAGAGACAG**GAGTACGATTGTACAAAGAATTAGAAAACCG**	**Multiplex 1 (M1)**
		Rev.	GTCTCGTGGGCTCGGAGATGTGTATAAGAGACAG**ATAACTCACTATCCCTATCTAAGAATATTC**	
PfK13.4		Fw.	TCGTCGGCAGCGTCAGATGTGTATAAGAGACAG**TAAGTGGAAGACATCATGTAACCAGAGA**	**Multiplex 2 (M2)**
		Rev.	GTCTCGTGGGCTCGGAGATGTGTATAAGAGACAG**CTTCTACATTCGGTATAATAGAAGAGCC**	
PfK13.5		Fw.	TCGTCGGCAGCGTCAGATGTGTATAAGAGACAG**ATGATGGCTCTTCTATTATACCGAATG**	**Multiplex 1 (M1)**
		Rev.	GTCTCGTGGGCTCGGAGATGTGTATAAGAGACAG**GCTATTAAAACGGAGTGACCAAATCTG**	
PfMSP2	PF3D7_0206800	Fw.	TCGTCGGCAGCGTCAGATGTGTATAAGAGACAG**TGCTTATAATATGAGTATAAGGAGAAGTATG**	**Multiplex 2 (M2)**
		Rev.	GTCTCGTGGGCTCGGAGATGTGTATAAGAGACAG**AGTGTCTGCATCTTGAGTGGGTGGAAC**	
Mito.1	Mitochondrion	Fw.	TCGTCGGCAGCGTCAGATGTGTATAAGAGACAG**CTTCCCTTCTCGCCATTTGATAGCGG**	**Multiplex 5 (M5)**
		Rev.	GTCTCGTGGGCTCGGAGATGTGTATAAGAGACAG**GAAAAAGGAATGAGTTTTGAAATCTCTAGTA**	
Mito.2		Fw.	TCGTCGGCAGCGTCAGATGTGTATAAGAGACAG**GATATGATAAATGTAAATACTCTGTAGTTTG**	**Simplex**
		Rev.	GTCTCGTGGGCTCGGAGATGTGTATAAGAGACAG**CCTGCATTAACATCATTATATGGTACATC**	
Mito.3		Fw.	TCGTCGGCAGCGTCAGATGTGTATAAGAGACAG**CAGTAATACTACGTACTGAATTATATTCTTC**	**Multiplex 5 (M5)**
		Rev.	GTCTCGTGGGCTCGGAGATGTGTATAAGAGACAG**GAAGACATAATACTAGCGACTCCAGATAC**	

Table S2. Index primers page 1/3

**Primer name**	**Primer sequence**
Index_1_F	AATGATACGGCGACCACCGAGATCTACACaaaagggaTCGTCGGCAGCGT
Index_2_F	AATGATACGGCGACCACCGAGATCTACACaaacagccTCGTCGGCAGCGT
Index_3_F	AATGATACGGCGACCACCGAGATCTACACaaactcgcTCGTCGGCAGCGT
Index_4_F	AATGATACGGCGACCACCGAGATCTACACaaagacacTCGTCGGCAGCGT
Index_5_F	AATGATACGGCGACCACCGAGATCTACACtttcctacTCGTCGGCAGCGT
Index_6_F	AATGATACGGCGACCACCGAGATCTACACtggttacaTCGTCGGCAGCGT
Index_7_F	AATGATACGGCGACCACCGAGATCTACACtgcaccatTCGTCGGCAGCGT
Index_8_F	AATGATACGGCGACCACCGAGATCTACACtctgagttTCGTCGGCAGCGT
Index_9_F	AATGATACGGCGACCACCGAGATCTACACacacatggTCGTCGGCAGCGT
Index_10_F	AATGATACGGCGACCACCGAGATCTACACacgtcttcTCGTCGGCAGCGT
Index_11_F	AATGATACGGCGACCACCGAGATCTACACaccacacaTCGTCGGCAGCGT
Index_12_F	AATGATACGGCGACCACCGAGATCTACACagagacacTCGTCGGCAGCGT
Index_13_F	AATGATACGGCGACCACCGAGATCTACACagcagtctTCGTCGGCAGCGT
Index_14_F	AATGATACGGCGACCACCGAGATCTACACaggctagaTCGTCGGCAGCGT
Index_15_F	AATGATACGGCGACCACCGAGATCTACACatcgtgtgTCGTCGGCAGCGT
Index_16_F	AATGATACGGCGACCACCGAGATCTACACatgaccgcTCGTCGGCAGCGT
Index_17_F	AATGATACGGCGACCACCGAGATCTACACatgggaagTCGTCGGCAGCGT
Index_18_F	AATGATACGGCGACCACCGAGATCTACACcacaggtgTCGTCGGCAGCGT
Index_19_F	AATGATACGGCGACCACCGAGATCTACACcagtactaTCGTCGGCAGCGT
Index_20_F	AATGATACGGCGACCACCGAGATCTACACcatgcttgTCGTCGGCAGCGT
Index_21_F	AATGATACGGCGACCACCGAGATCTACACccacagaaTCGTCGGCAGCGT
Index_22_F	AATGATACGGCGACCACCGAGATCTACACcccttgctTCGTCGGCAGCGT
Index_23_F	AATGATACGGCGACCACCGAGATCTACACcctaatacTCGTCGGCAGCGT
Index_24_F	AATGATACGGCGACCACCGAGATCTACACcgactgttTCGTCGGCAGCGT
Index_25_F	AATGATACGGCGACCACCGAGATCTACACcgctgttcTCGTCGGCAGCGT
Index_26_F	AATGATACGGCGACCACCGAGATCTACACcgcttcagTCGTCGGCAGCGT
Index_27_F	AATGATACGGCGACCACCGAGATCTACACctcggcttTCGTCGGCAGCGT
Index_28_F	AATGATACGGCGACCACCGAGATCTACACctgcacgtTCGTCGGCAGCGT
Index_29_F	AATGATACGGCGACCACCGAGATCTACACcttgctcaTCGTCGGCAGCGT
Index_30_F	AATGATACGGCGACCACCGAGATCTACACgatgtcagTCGTCGGCAGCGT
Index_31_F	AATGATACGGCGACCACCGAGATCTACACgataccctTCGTCGGCAGCGT
Index_32_F	AATGATACGGCGACCACCGAGATCTACACgatccaacTCGTCGGCAGCGT
Index_33_F	AATGATACGGCGACCACCGAGATCTACACgctaggttTCGTCGGCAGCGT
Index_34_F	AATGATACGGCGACCACCGAGATCTACACgcgtgtcaTCGTCGGCAGCGT
Index_35_F	AATGATACGGCGACCACCGAGATCTACACgcactagtTCGTCGGCAGCGT
Index_36_F	AATGATACGGCGACCACCGAGATCTACACggtcttaaTCGTCGGCAGCGT
Index_37_F	AATGATACGGCGACCACCGAGATCTACACggctcataTCGTCGGCAGCGT
Index_38_F	AATGATACGGCGACCACCGAGATCTACACggttgctgTCGTCGGCAGCGT

Table S2. Index primers page 2/3

Index_39_F	AATGATACGGCGACCACCGAGATCTACACgtgcgagaTCGTCGGCAGCGT
Index_40_F	AATGATACGGCGACCACCGAGATCTACACgtctgtggTCGTCGGCAGCGT
Index_41_F	AATGATACGGCGACCACCGAGATCTACACgtggagatTCGTCGGCAGCGT
Index_42_F	AATGATACGGCGACCACCGAGATCTACACtaaccacgTCGTCGGCAGCGT
Index_43_F	AATGATACGGCGACCACCGAGATCTACACtagcgttgTCGTCGGCAGCGT
Index_44_F	AATGATACGGCGACCACCGAGATCTACACtagtgggaTCGTCGGCAGCGT
Index_45_F	AATGATACGGCGACCACCGAGATCTACACtcctaacgTCGTCGGCAGCGT
Index_46_F	AATGATACGGCGACCACCGAGATCTACACtcgttcacTCGTCGGCAGCGT
Index_47_F	AATGATACGGCGACCACCGAGATCTACACtgatcgacTCGTCGGCAGCGT
Index_48_F	AATGATACGGCGACCACCGAGATCTACACttcatggtTCGTCGGCAGCGT
Index_49_F	AATGATACGGCGACCACCGAGATCTACACtttgacgcTCGTCGGCAGCGT
Index_50_F	AATGATACGGCGACCACCGAGATCTACACttcgcagaTCGTCGGCAGCGT
Index_1_R	CAAGCAGAAGACGGCATACGAGATtcccttttGTCTCGTGGGCTCGGAGA
Index_2_R	CAAGCAGAAGACGGCATACGAGATggctgtttGTCTCGTGGGCTCGGAGA
Index_3_R	CAAGCAGAAGACGGCATACGAGATgcgagtttGTCTCGTGGGCTCGGAGA
Index_4_R	CAAGCAGAAGACGGCATACGAGATgtgtctttGTCTCGTGGGCTCGGAGA
Index_5_R	CAAGCAGAAGACGGCATACGAGATgtaggaaaGTCTCGTGGGCTCGGAGA
Index_6_R	CAAGCAGAAGACGGCATACGAGATtgtaaccaGTCTCGTGGGCTCGGAGA
Index_7_R	CAAGCAGAAGACGGCATACGAGATatggtgcaGTCTCGTGGGCTCGGAGA
Index_8_R	CAAGCAGAAGACGGCATACGAGATaactcagaGTCTCGTGGGCTCGGAGA
Index_9_R	CAAGCAGAAGACGGCATACGAGATccatgtgtGTCTCGTGGGCTCGGAGA
Index_10_R	CAAGCAGAAGACGGCATACGAGATgaagacgtGTCTCGTGGGCTCGGAGA
Index_11_R	CAAGCAGAAGACGGCATACGAGATtgtgtggtGTCTCGTGGGCTCGGAGA
Index_12_R	CAAGCAGAAGACGGCATACGAGATgtgtctctGTCTCGTGGGCTCGGAGA
Index_13_R	CAAGCAGAAGACGGCATACGAGATagactgctGTCTCGTGGGCTCGGAGA
Index_14_R	CAAGCAGAAGACGGCATACGAGATtctagcctGTCTCGTGGGCTCGGAGA
Index_15_R	CAAGCAGAAGACGGCATACGAGATcacacgatGTCTCGTGGGCTCGGAGA
Index_16_R	CAAGCAGAAGACGGCATACGAGATgcggtcatGTCTCGTGGGCTCGGAGA
Index_17_R	CAAGCAGAAGACGGCATACGAGATcttcccatGTCTCGTGGGCTCGGAGA
Index_18_R	CAAGCAGAAGACGGCATACGAGATcacctgtgGTCTCGTGGGCTCGGAGA
Index_19_R	CAAGCAGAAGACGGCATACGAGATtagtactgGTCTCGTGGGCTCGGAGA
Index_20_R	CAAGCAGAAGACGGCATACGAGATcaagcatgGTCTCGTGGGCTCGGAGA
Index_21_R	CAAGCAGAAGACGGCATACGAGATttctgtggGTCTCGTGGGCTCGGAGA
Index_22_R	CAAGCAGAAGACGGCATACGAGATagcaagggGTCTCGTGGGCTCGGAGA
Index_23_R	CAAGCAGAAGACGGCATACGAGATgtattaggGTCTCGTGGGCTCGGAGA
Index_24_R	CAAGCAGAAGACGGCATACGAGATaacagtcgGTCTCGTGGGCTCGGAGA
Index_25_R	CAAGCAGAAGACGGCATACGAGATgaacagcgGTCTCGTGGGCTCGGAGA
Index_26_R	CAAGCAGAAGACGGCATACGAGATctgaagcgGTCTCGTGGGCTCGGAGA

Table S2. Index primers page 3/3

Index_27_R	CAAGCAGAAGACGGCATACGAGATaagccgagGTCTCGTGGGCTCGGAGA
Index_28_R	CAAGCAGAAGACGGCATACGAGATacgtgcagGTCTCGTGGGCTCGGAGA
Index_29_R	CAAGCAGAAGACGGCATACGAGATtgagcaagGTCTCGTGGGCTCGGAGA
Index_30_R	CAAGCAGAAGACGGCATACGAGATctgacatcGTCTCGTGGGCTCGGAGA
Index_31_R	CAAGCAGAAGACGGCATACGAGATagggtatcGTCTCGTGGGCTCGGAGA
Index_32_R	CAAGCAGAAGACGGCATACGAGATgttggatcGTCTCGTGGGCTCGGAGA
Index_33_R	CAAGCAGAAGACGGCATACGAGATaacctagcGTCTCGTGGGCTCGGAGA
Index_34_R	CAAGCAGAAGACGGCATACGAGATtgacacgcGTCTCGTGGGCTCGGAGA
Index_35_R	CAAGCAGAAGACGGCATACGAGATactagtgcGTCTCGTGGGCTCGGAGA
Index_36_R	CAAGCAGAAGACGGCATACGAGATttaagaccGTCTCGTGGGCTCGGAGA
Index_37_R	CAAGCAGAAGACGGCATACGAGATtatgagccGTCTCGTGGGCTCGGAGA
Index_38_R	CAAGCAGAAGACGGCATACGAGATcagcaaccGTCTCGTGGGCTCGGAGA
Index_39_R	CAAGCAGAAGACGGCATACGAGATtctcgcacGTCTCGTGGGCTCGGAGA
Index_40_R	CAAGCAGAAGACGGCATACGAGATccacagacGTCTCGTGGGCTCGGAGA
Index_41_R	CAAGCAGAAGACGGCATACGAGATatctccacGTCTCGTGGGCTCGGAGA
Index_42_R	CAAGCAGAAGACGGCATACGAGATcgtggttaGTCTCGTGGGCTCGGAGA
Index_43_R	CAAGCAGAAGACGGCATACGAGATcaacgctaGTCTCGTGGGCTCGGAGA
Index_44_R	CAAGCAGAAGACGGCATACGAGATtcccactaGTCTCGTGGGCTCGGAGA
Index_45_R	CAAGCAGAAGACGGCATACGAGATcgttaggaGTCTCGTGGGCTCGGAGA
Index_46_R	CAAGCAGAAGACGGCATACGAGATgtgaacgaGTCTCGTGGGCTCGGAGA
Index_47_R	CAAGCAGAAGACGGCATACGAGATgtcgatcaGTCTCGTGGGCTCGGAGA
Index_48_R	CAAGCAGAAGACGGCATACGAGATaccatgaaGTCTCGTGGGCTCGGAGA
Index_49_R	CAAGCAGAAGACGGCATACGAGATgcgtcaaaGTCTCGTGGGCTCGGAGA
Index_50_R	CAAGCAGAAGACGGCATACGAGATtctgcgaaGTCTCGTGGGCTCGGAGA

Table S3. qPCR primers

Primer name	Primer sequence
Pfdhfr.1_qPCR_F	GTC TGC GAC GTT TTC GAT ATT TAT G
Pfdhfr.1_qPCR_R	TCC ATG GTA ATA CTC CTT TAT TTC CTA
Pfmdr1.1_qPCR_F	GAT GGT AAC CTC AGT ATC AAA G
Pfmdr1.1_qPCR_R	GTT GTG CAG GTA AAC ATT TAA ACG G
Pfmdr1.7_qPCR_F	GCG TGT ATT TGC TGT AAG AGC TAG
Pfmdr1.7_qPCR_R	GGA TCT TTA AAC ATT TCA TCA TCT GAA C
Pfmdr1.8_qPCR_F	CTA CAG CAA TCG TTG GAG AAA CAG
Pfmdr1.8_qPCR_R	GCA GAT CCA GAT TGG TTT GAA AAT TC
Pfcrt.2_qPCR_F	GTG GAG GTT CTT GTC TTG GTA AAT
Pfcrt.2_qPCR_R	TTC GGA TGT TAC AAA ACT ATA GTT ACC
Pfdhps.3_qPCR_F	TAC AAC ACA CAG ATA TAG CAT ACT TTT A
Pfdhps.3_qPCR_R	GAA ATT CTA TCT TTT AAT ACA TAT ATC CTT T
Pfdhps.4_qPCR_F	AGT GTA GTT CTA ATG CAT AAA AGA GG
Pfdhps.4_qPCR_R	CCT AAT CCA ATA TCA AAT AGT ATC C
PfK13.1_qPCR_F	GAT AGG GAA TCT GGT GGT AAC AGC
PfK13.1_qPCR_R	CAA AGT TCG AAT CTA ATA CAC TCA T
PfK13.2_qPCR_F	TAC GAT TGT ACA AAG AAT TAG AAA ACC
PfK13.2_qPCR_R	CAT CAA ATC GTT TCC TAT GTT CTT C
PfK13.3_qPCR_F	GTT GAT GCA AAT ATT GCT ACT GAA A
PfK13.3_qPCR_R	CTA AGA ATA TTC TTC CTT GTT TAT CTC T
PfK13.4_qPCR_F	GTC AAC AAT GCT GGC GTA TGT
PfK13.4_qPCR_R	CAT CTC TTA AAC GAT CAT ACA CCT
PfK13.5_qPCR_F	CAA TTT CCA TAT GCC TTA TTA GAA GCT
PfK13.5_qPCR_R	CTC TGG TAC ACC ATT TAG AAA TTG C
PfMSP.2_qPCR_F	GTA CCT CTT CAG AAA ATC CAA ATC AT
PfMSP.2_qPCR_R	GGG TGG AAC ATT TGA TTT AGT TTG AG
Mito.1_qPCR_F	GAA TAA GAA CTC TAT AAA TAA CCA GAC TA
Mito.1_qPCR_R	ATA TAT GAT ACT TCT ACC GAA TGG TTT A
Mito.2_qPCR_F	ATG GAT ATG GTG ATA AAC TAA AAT GTA ATA
Mito.2_qPCR_R	CTT TTC TGT AGG GAT ATT ATT TAC ATT TA
Mito.3_qPCR_F	ACC AAA TCC TCC GAA TAA TCC TGG CA
Mito.3_qPCR_R	TCC ATC CAG TTC CAC CAC CAA ATT CTG
